# Identifying the safe operating space for food systems

**DOI:** 10.1038/s43016-025-01252-6

**Published:** 2025-10-31

**Authors:** Sofie te Wierik, Fabrice DeClerck, Arthur Beusen, Dieter Gerten, Federico Maggi, Anna Norberg, Kevin Noone, Lena Schulte-Uebbing, Marco Springmann, Fiona H. M. Tang, Wim de Vries, Detlef van Vuuren, Sonja Vermeulen, Johan Rockström

**Affiliations:** 1 Potsdam Institute for Climate Impact Research (PIK), Member of the Leibniz Association, Potsdam, Germany; 2 PBL Netherlands Environmental Assessment Agency, The Hague, Netherlands; 3 EAT, Oslo, Norway; 4 Alliance of Biodiversity and CIAT, CGIAR, Montpellier, France; 5 Department of Earth Sciences-Geochemistry, Utrecht University, Utrecht, Netherlands; 6 Geography Department, Humboldt-Universität zu Berlin, Berlin, Germany; 7 The University of Sydney, Sydney, New South Wales Australia; 8 Stockholm University, Stockholm, Sweden; 9 Institute for Global Health, University College London, London, UK; 10 Department of Civil Engineering, Monash University, Clayton, Victoria, Australia; 11 Wageningen University and Research, Wageningen, Netherlands; 12 Copernicus Institute of Sustainable Development, Utrecht University, Utrecht, Netherlands; 13 Senior Advisor at Clim-Eat. Agro Business Park 10, Wageningen, Netherlands; 14 Institute of Environmental Science and Geography, University of Potsdam, Potsdam, Germany; 15 Stockholm Resilience Centre, Stockholm University, Stockholm, Sweden

**Keywords:** Sustainability, Environmental impact

## Abstract

Global environmental pressures from food systems threaten biodiversity and the stability of the Earth system, yet the safe operating space for food systems is unknown. Here we calculate food system boundaries as shares of planetary boundaries, proposing budgets for the food system across nine boundaries. Our results indicate that food systems are a critical driver of planetary boundary transgressions, dominating at least four transgressed boundaries (that is, biosphere integrity, land system change, freshwater change and biogeochemical flows) while strongly contributing to the transgression of two more (that is, climate change and novel entities). Moreover, global food systems are currently beyond all nine food system boundaries; moving to the safe operating space requires reducing related greenhouse gas emissions substantially, halting the conversion of intact nature to agriculture, redistributing fertilizer inputs, limiting pesticide and antibiotic use, and preserving critical freshwater flows without negatively affecting yields.

## Abstract (web summary)

Consensus exists on the urgent need for food systems to be more sustainable, but defining their environmentally safe operating space is challenging. This study proposes food system boundaries as a share of planetary boundaries, defining budgets across nine boundaries and revealing where boundary transgression is most critical.

## Main

The planetary boundary (PB) framework^1^ aims to define and quantify a safe operating space for humanity based on environmental thresholds, within which human activities can unfold without threatening the stability of the Earth system^1^. As of now, six of nine PBs are being transgressed^2^. There is ample evidence that food systems are a dominant driver of global environmental change^3–5^, mainly from agricultural production^6^. Several studies quantifying the impacts of agricultural production^6,7^ and food systems^5^ across the PBs concordantly show that agricultural production alone is responsible for the current transgression of the biosphere integrity and biogeochemical flows PBs^6^ and contributes strongly to the transgression of other PBs, thereby pushing the Earth system further into a zone of increasing risk. Dietary shifts, changes in production systems, improved land governance, and reduction in food loss and waste can help bring such systems back to the safe operating space defined by PBs for freshwater, biogeochemical flows, land-system change, biosphere integrity and climate change, while still producing sufficient food to feed around 10 billion people in 2050^5,7^. Yet, estimating the full potential of food systems to move back into this safe operating space is a complex exercise which, so far, remains limited to a subset of the boundaries^7–9^ or uses global proxy indicators for PB domains^5,6^. In addition, some of these studies lack the regional detail necessary to assess transgression risks for local ecosystems^10^. In other words, a framework to navigate food systems back into their safe operating space is needed, but a comprehensive assessment of the boundaries for the food system is still lacking.

**Fig. 1:**
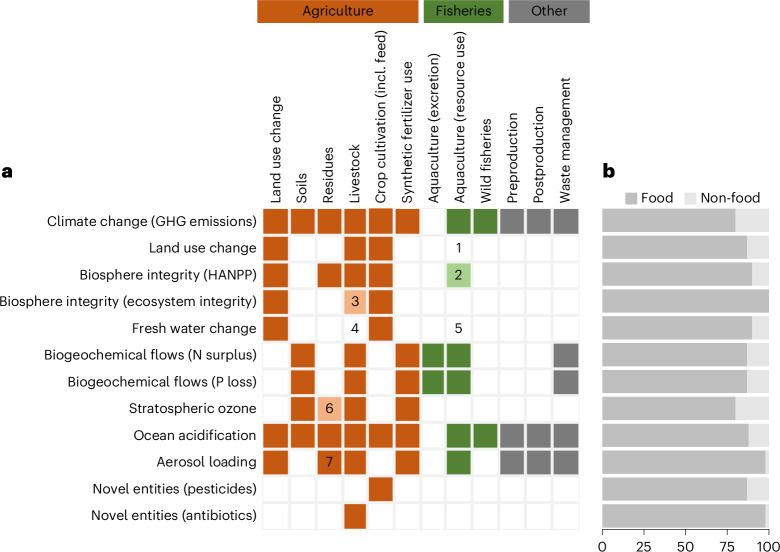
Definition of the food system and inclusion of food system elements and non-food agricultural production. **a**, Elements (columns) of the food system that are included in our estimate of the contribution of the food system to the PBs (rows). A further breakdown of elements is given in Supplementary Table 12. Elements within ‘Agriculture’ (orange) are equal to IPCC AFOLU accounting. Light shaded boxes represent elements that are partly included. Box numbers: 1, excludes surface area of ponds (only covers 0.055 Mkm^2^ (ref. ^82^); 2, includes crop feed but excludes wild fish feed; 3, includes pasture lands but excludes seminatural extensive grazing land; 4, excludes livestock drinking water (only 2% of livestock water use)^83^; 5, evaporation from ponds is excluded (no data); 6, higher end of range includes biomass burning; 7, includes biomass burning from vegetation clearance in forest and peatlands. **b**, Estimated percentage share of non-food agricultural production (that is, crop and animal production for fibre, fuel or other industrial uses) included in our presented food system contribution estimates in Table 1. See Supplementary Table 1 and the data repository for Fig. 1 for details.

In this study, we consolidate scientific evidence on the impact of food systems across all nine planetary boundaries, proposing a set of food system boundaries (FSBs) that may serve as a guideline to ensure that food systems operate within their safe operating space (Table 1). FSBs correspond to shares of PBs estimated specifically for food systems, calculated as scenario-based budgets for the food system. We account for the impacts of food production (consistent with the ‘agriculture, forestry and land use’ (AFOLU) category used in IPCC reports) and pre- and postprocessing stages, although our calculations are sometimes limited by data granularity (for example, we were not always able to disaggregate food versus non-food agricultural production) or availability (for example, no estimates were available for pre- and postprocessing impacts). First, we quantified the present-day contribution of food systems to the PBs, updating existing calculations^6^ based on a consistent methodological approach across all PB domains (see Methods) and guided by the most recent advances in PB science^2,11^, thereby aiming to use robust evidence from multimodel ensembles. Despite the data limitations mentioned above, evidence suggests that the non-food contribution of agricultural production to each boundary is minor at the global level (Fig. 1). Second, we identified FSBs by allocating a share of the safe operating space to food systems based on available scientific evidence. In contrast to the PBs—which are based on quantified levels of risk associated with global change^1^—FSBs are based on several principles adopted in current scientific assessments, ranging from (economically) optimized allocations of emission reductions using integrated assessment models (for example, as for the climate change FSB), to proportional reductions based on their current contributions to boundary transgressions (for example, for freshwater and biogeochemical flow FSBs, assuming similar efforts across sectors to enable a return to the safe operating space), to effects emerging from boundary interactions (for example, for ozone depletion and aerosol loading FSBs, responding to reductions in nitrogen surplus under the biogeochemical flow FSBs). There is no single, uniform approach available to quantify the FSBs across the current literature; for transparency, a full overview of studies included is provided in Supplementary Table 1.

**Table 1:** Overview of the state of food systems and FSBs across planetary boundaries

Earth system domain	Control variable	PB	Current state	Contribution of the food system (% of total)	FSB	FSB status
Climate change	Atmospheric CO_2_ concentration (ppm CO_2_)	350 ppm	419 ppm	16–17.7 GtCO_2_e yr^−1^ (∼30% of total emissions)	<5 GtCO_2_e yr^−1^ of remaining emissions	Transgressed
	Total anthropogenic radiative forcing at top-of-atmosphere (W m^−2^)	+1.2 W m^−2^	+2.91 W m^−2^	+0.69 W m^−2^ (2% of total radiative forcing)		
Land system change	Global: Area of intact land as the percentage of original cover ^a^	Global: 50–60% remaining intactness	Global: 50% remaining intactness	Global: 48 Mkm^2^ (37% of total land area)	Agricultural land <48 Mkm^2^ (halting conversion of intact land); <40–50% agricultural land at ecoregion level; restoring 8.5 Mkm^2^ in forest ecoregions	Partly transgressed
	Forest biome: Area of forested land as the percentage of potential forest (% area remaining)	Forest biomes: Tropical, 85%; temperate, 50%; boreal, 85%	Forest biomes: Tropical, 37.5–83.9%; temperate, 34.2–51.2%; boreal, 56.6–70.3%	Forest biomes: Agricultural area covers 25–50% of tropical forest, 20–65% of temperate forest and 3% of boreal forest		
	Ecoregions: Area of intact land as a percentage of the original cover by ecoregion^a^	Ecoregions: 50–60% remaining intact across all ecoregions	Ecoregions: 10–95% remaining intact	Ecoregions: 34% of ecoregions below the intactness threshold (50%) due to agriculture alone		
Biosphere integrity	Ecosystem functional integrity^a^	>20–25% habitat per km^2^ for supporting agroecosystem functioning	30–60% of agricultural lands below boundary	88% of agricultural lands used for food production	100% of agricultural lands for food production within boundary	Transgressed
	Biosphere functional integrity	HANPP ^1^ < 10% of Holocene NPP, that is, remaining >90% for the biosphere	13–16.8 GtC yr^−1^ (25–30% of Holocene NPP)	9.9–11.7 GtC yr^−1^ (72–85% of total HANPP)	<5.5 GtC yr^−1^	Transgressed
Freshwater use	Consumptive blue water use (km^3^ yr^−1^)^d^	2,800 km^3^ yr^−1^	1,800–2,600 km^3^ yr^−1^	>1,200–1,800 km^3^ yr^−1^ (70% of consumptive use)	< 2,000 km^3^ yr^−1^	Regionally transgressed
	Green water (% of ice-free land area beyond 5–95th variability envelope)	11.1% of ice-free land area with local deviations	15.8% of ice-free land area with local deviations	16.8% of agricultural land is beyond local variability envelope	Agricultural land remains within global soil moisture variability envelopes (11.1% of ice-free land area beyond variability envelope)	Transgressed
Biogeochemical glows	Nitrogen surplus (TgN yr^−1^)^b^	57 TgN yr^−1^	119 TgN yr^−1^	50%, 70% and 80% to deposition, surface water load, and groundwater leaching, respectively	<57 TgN yr^−1^ (corresponding to agricultural nitrogen input <134 TgN yr^−1^ based on current NUE)	Transgressed
	Phosphorus loss to surface water (TgP yr^−1^)^c^	6.1 TgP yr^−1^	9.7 TgP yr^−1^	7.2 TgP yr^−1^ (75% of total phosphorus delivery)	<4.6 TgP yr^−1^	Transgressed
Stratospheric ozone depletion	Stratospheric O_3_ concentration (global average) (Dobson unit, DU)	<5% reduction from preindustrial level assessed by latitude (∼276 DU)	284 DU	3.9–4.2 TgN yr^−1^ agricultural N_2_O emissions as main ozone-depleting substance (54–69% of total N_2_O emissions)	<1.8 TgN yr^−1^	Transgressed although globally within boundary
Aerosol loading	Interhemispheric difference in AOD	<0.1 (mean annual interhemispheric difference)	0.076	Northern hemisphere: >80% of NH_3_ emissions forming secondary PM2.5 concentrations Southern hemisphere: >50% of PM2.5 from biomass burning due to food system	Northern hemisphere: <20 TgNH_3_ (45% reduction in global NH_3_ emissions compared with current emissions) Southern hemisphere: halting biomass burning emissions from land conversion	Transgressed
Ocean acidification	Average global surface ocean saturation state with respect to aragonite (*Ω*_arag_)	≥80% of the preindustrial averaged global *Ω*_arag_ of 3.4	2.8	25% of CO_2_ emissions as main driver of change in *Ω*_arag_	Zero net CO_2_ emissions from land use change and fossil emissions in the food chain	Transgressed
Novel entities	Percentage of synthetic chemicals released to the environment without adequate safety testing^e,f^	-	-	PAS application (Tg yr^−1^): 3.3–3.7 (85–90% of total pesticide use)	<1 TgPAS yr^−1^ (>70% reduction of current application) to avoid high pollution risk; <0.2 TgPAS yr^−1^ (>90% reduction of current application) to remain below low pollution risk	Transgressed
		-	-	Antimicrobial use in food animals (t yr^−1^): 73–130 kt yr^−1^ (73% of total antimicrobial use)	Halting prophylactic use and reducing overall use by >50% (max. 36,500-75,000 t yr^−1^)	Transgressed

The current contributions of the food system to the PBs are provided in absolute numbers and relative contributions (in brackets). The control variable, PB and current state are provided primarily based on the most recent assessments by ref. ^2^, unless indicated otherwise: ^a^ref. ^11^, ^b^ref. ^10^, ^c^ref. ^35^, ^d^ref. ^9^, ^e^ref. ^57^, ^f^ref. ^59^. Note that atmospheric CO_2_ concentration control variable under the climate change boundary is expressed in CO_2_e for the food system contribution and the FSB (including non-CO_2_ gasses of CH_4_ and N_2_O). ‘Partly transgressed’ under ‘FSB status’ suggests either that at least one out of several control variables is transgressed; or that thresholds are breached regionally but remain within PBs globally.

**Fig. 2:**
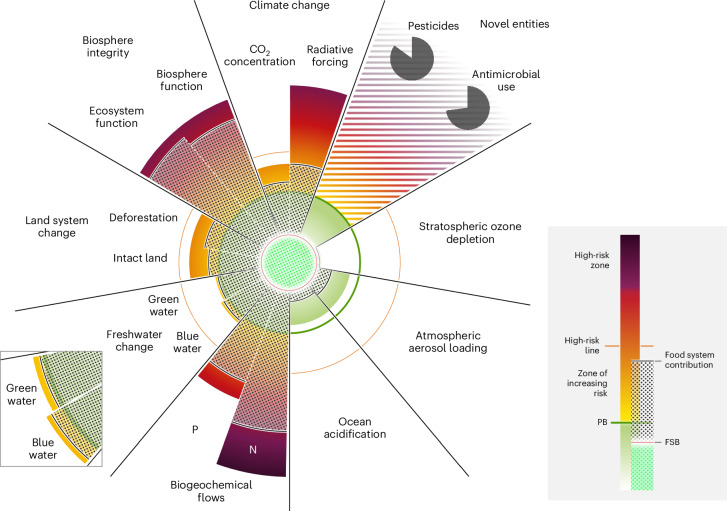
Status of the food system across PBs and the FSBs. FSBs (pink line) are represented in a stylized and uniform radius within the safe operating space (green sphere). The radar plot is adapted from ref. ^2^ (data) and ref. ^11^ (visualization). The contribution of the food system (in percentages, see Table 1 and Supplementary Table 11, indicated by the black dotted pattern) is projected based on the length of each wedge starting from the PB (for all transgressed boundaries) and the FSB (for the other three boundaries). The components representing novel entities (pesticides and antimicrobial use) are shown as pie charts within the largest set of all (up to now unquantified) novel entities. Note that CO_2_ concentration is provided here in terms of CO_2_, in contrast to the CO_2_e in Table 1. Credit: Azote.

In summary, this study aims to provide an integrated assessment of the safe operating space for food systems (Fig. 2). Because food systems impact multiple planetary boundaries through local-to-regional practices on land and in marine systems, we opted to retain the biogeophysical and global character of the PB framework while also considering more explicitly regional-scale processes and impacts across the different PBs.

## Results

### Climate change

Food is responsible for around 30% of current greenhouse gas (GHG) emissions. AFOLU emissions amount to 11.9 GtCO_2_e yr^−1^ (21% of total emissions), comprising CO_2_ emissions from land conversion (5.9 GtCO_2_e yr^−1^), methane (CH_4_) emissions (4.2 GtCO_2_e yr^−1^) and nitrous oxide (N_2_O) emissions (1.8 GtCO_2_e yr^−1^). Other emissions (energy use on-farm and post-farmgate, transport, cold storage, processing, retail, catering, food management in homes, and waste) amount 5.8 GtCO_2_e yr^−1^ (ref. ^12^) (Supplementary Table 2 and Supplementary Text 1). These emissions have added an estimated ±1 W m^−2^ of positive radiative forcing with respect to preindustrial levels. They are partly offset by negative forcing from agricultural-induced aerosol emissions and land use change, suggesting a contribution to the net radiative forcing effect of 24% (although large uncertainties for some forcers exist) (Supplementary Table 4). Various studies—including top-down economically optimized allocation modelling using integrated assessment models (IAMs), and bottom-up sectoral mitigation potential assessments—confirm that AFOLU emissions should be reduced to around 5 GtCO_2_e yr^−1^ (Supplementary Table 3), comprising solely non-CO_2_ emissions from food systems that are hard to abate, to remain within 1.5 °C of warming if combined with carbon removal strategies^13^. We therefore adhere to 5 GtCO_2_e yr^−1^ as the FSB for climate change. Note that, in addition, negative CO_2_ emissions from AFOLU are critical across all 1.5 °C scenarios by 2050^13^, which are not included in the FSB as defined here.

### Land system change

**Fig. 3:**
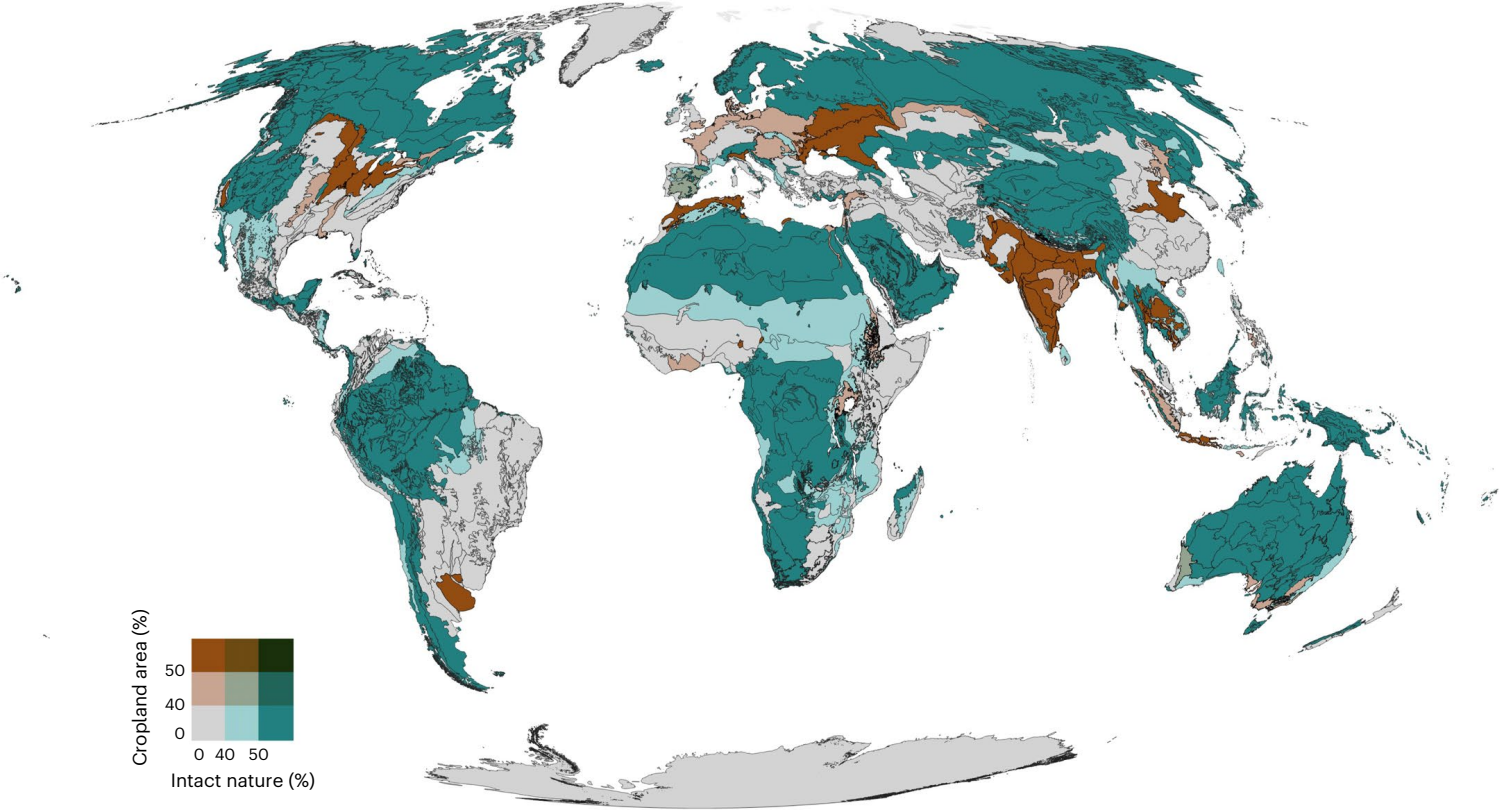
Exceedance of intact nature by cropland. Intactness data (blue) represents the land where natural processes predominate^11,18^. Cropland (brown) data are based on HYDE3.2 for 2017^84^. In 10% of the ecoregions, cropland alone is already covering >40% of the land area. Including grazing land increases the number of ecoregions that are in the zone of increasing risk from 10% to 43% (Supplementary Fig. 2).

Agricultural land covers 48 Mkm^2^, which is 37% of the global land area. A third of agricultural land is used for crop production (16 Mkm^2^), while the remaining 32 Mkm^2^ comprises permanent meadows and pastures^14^. Continuing agricultural expansion predominantly occurs at the expense of tropical forest^15,16^, making food systems the most important driver of deforestation and loss of intact nature^17^. Various studies confirm that 50–60% of land must remain intact to protect biodiversity and its contributions to climate mitigation and hydrological cycles^11,18,19^. We adopt this (lower) bound of 50% of remaining intactness as boundaries at both the global and ecoregion level, with particular attention to forest biomes for climate regulation^2^. Distribution of agricultural land and remaining intactness is irregular across ecoregions (Fig. 3) and forest biomes (Supplementary Fig. 1). In 5% of the ecoregions, croplands alone breach the 50% intactness boundary. When including grazing lands, 34% of ecoregions are transgressed (Supplementary Text 2). Staying within the land system PB requires zero conversion of remaining intact ecosystems, while restoring intactness in areas with substantial losses (Fig. 3).

### Biosphere integrity

Land conversion, freshwater drawdown, and pollution from chemical and nutrient overloads^20^ in agriculture are the main drivers of biodiversity loss. They affect the supporting functions across agricultural ecosystems (that is, ecosystem integrity, expressed as the embedded natural habitat on agricultural lands) and the global energy balance of the biosphere (that is, biosphere integrity, expressed as the human-appropriated net primary productivity (HANPP)). Ecosystem functional integrity requires (semi)natural habitat embedded in agricultural lands (for example, hedgerows, buffer strips) to preserve fine-scaled ecosystem functions (for example, pollination, pest control, sediment and nutrient capture functions). Recent analyses support 10–25% (semi)natural habitat per km^2^ as a critical minimum value below which such functions are lost^11,21,22^, which we adopt as a boundary for all agricultural lands (of which 88% are used to produce food)^4^. Between 33% and 60% of agricultural lands are currently below the boundary^22^.

The PB for biosphere integrity (HANPP) suggests that 90% of Holocene NPP (in total 55 GtC yr^−1^), plus the climate resilience response of the biosphere (>15 GtC yr^−1^ additional uptake from CO_2_ fertilization), should remain available for ecosystems^2^, implying that around 10% of Holocene NPP (5.5 GtC yr^−1^) can be safely appropriated. Food systems appropriate NPP via land conversion and harvesting of biomass, in total amounting to 9.9–11.7 GtC yr^−1^ (72–85% of total HANPP; Supplementary Table 4)^2,23,24^. Considering the dominant food system share, we propose that the FSB is placed at the PB (<10% HANPP, or 5.5 GtC yr^−1^), while ensuring that the Earth system does not exceed the upper end of the zone of increasing risk (20% HANPP)^2^ when including additional land uses (for example, urbanization, industrial use).

### Freshwater change

Most human freshwater consumption from water withdrawals is for irrigating crops. Process-based models estimate irrigation consumptive use at around 1,200 km^3^ yr^−1^ (of which 545 km^3^ yr^−1^ is sourced from groundwater)^25^, while water footprint assessments appear to be at the higher end of the range (1,800 km^3^ yr^−1^)^5^ (Supplementary Table 6). However, as the majority of agricultural lands is rainfed, accounting for consumption of green water (plant-available soil moisture) in addition to blue water consumption adds another ∼7,000 km^3^ yr^−1^ (refs. ^26–28^). The PB for freshwater was initially expressed only in terms of a blue water consumptive use limit to preserve ecological flow requirements (EFRs)^1,29^. Recent scientific updates recognize green water and express the PB based on the global land area (as a percentage) that experiences significant wet or dry soil moisture (green water) or streamflow (blue water) events compared with preindustrial variability baselines^2,30,31^. Currently 18.2% (15.8%) of ice-free land is experiencing such local blue (green) deviations from preindustrial conditions, compared with 10.2% (11.1%) in the preindustrial period, indicating strong transgression of the freshwater change PB.

Quantifying the contributions of food systems and defining a FSB for blue and green water using this novel freshwater PB is challenging (Supplementary Text 4). We propose that food systems remain within local EFR boundaries for blue water, with a global budget of 2,000 km^3^ yr^−1^ (Supplementary Text 4), including groundwater^11^, while remaining within the global green water deviation variability envelope across all agricultural lands (11.1%; Supplementary Fig. 3). Although this suggests we are within the PB for blue water, irrigation alone is transgressing regional EFR limits at river basin level, summing up to a global water withdrawal overshoot of ∼1,000 km^3^ yr^−1^ (ref. ^32^). In addition, 16.8% of agricultural land is beyond local variability envelopes for green water, and well above the global preindustrial value of 11.1% (Supplementary Fig. 3)

### Biogeochemical flows

Nitrogen and phosphorus are key nutrients for crop growth and limited availability has adverse effects on crop yields. However, elevated nitrogen and phosphorus inputs in agriculture increase their losses to surface water, affecting aquatic biodiversity, causing harmful algal blooms and, in extreme cases, creating dead zones in coastal waters. Nitrogen losses to air cause terrestrial biodiversity loss from enhanced nitrogen deposition^33,34^, and add climatic forcing through N_2_O emissions. For nitrogen, we use regional nitrogen surplus boundaries derived by ref. ^10^, aggregated to a planetary nitrogen surplus, instead of the human-induced biological and chemical nitrogen fixation^2^. For phosphorus, we propose to use the phosphorus delivery (input) to surface water from agriculture, aquaculture and wastewater, with the losses from agriculture being dominated by soil phosphorus erosion (see Supplementary Text 5 for details on the control variables).

### Nitrogen

**Fig. 4:**
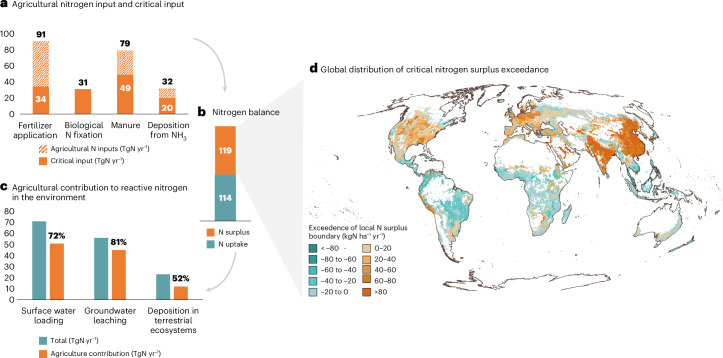
Agricultural nitrogen surplus. **a**–**d**, Global nitrogen input and critical input levels (where current input exceeds local thresholds) (**a**) and associated nitrogen uptake and surplus (**b**) that lead to exceedance of critical surplus value based on surface water loading, groundwater leaching and deposition in terrestrial ecosystems (**c**), and the spatial distribution of the critical nitrogen surplus exceedance (**d**). Agricultural inputs in **a** are provided for fertilizer application, biological nitrogen fixation, manure, and deposition from NH_3_. Numbers on top of the bars in **a** represent current inputs; the solid parts of the bars represent the required input reductions considering critical input values, assuming other nitrogen inputs will not change. Of the total inputs (233 TgN yr^−1^), 114 TgN yr^−1^ is taken up by plants while 119 TgN yr^−1^ is surplus. Data from refs. ^10,85^. See Supplementary Table 8 for details.

The nitrogen input on agricultural lands (in total 233 TgN yr^−1^ in 2010) derives from fertilizer, biological nitrogen fixation, manure and nitrogen (NH_3_ and NO_*x*_) deposition. Around half of the input is taken up by plants, while 119 TgN yr^−1^ remains as agricultural surplus which, together with other nitrogen sources, ends up in surface water (70 TgN yr^−1^), leaches to groundwater (56 TgN yr^−1^) and is deposited on terrestrial ecosystems (20 TgN yr^−1^). Food systems (including agriculture, aquaculture and nitrogen from wastewater) contribute around 70%, 80% and 50% to these processes, respectively (Supplementary Table 8), exceeding critical nitrogen surplus levels in two-thirds of the land area (Fig. 4). Respecting local nitrogen thresholds for surface water concentrations, groundwater leaching and deposition on terrestrial ecosystems, while allowing increased nitrogen input in nitrogen-deficient regions, leads to a nitrogen surplus boundary of 57 TgN yr^−1^. Proportional reduction (that is, assuming proportional reductions across sectors) suggests that agricultural nitrogen surplus is brought back from 119 TgN yr^−1^ to the PB value of 57 TgN yr^−1^, associated with an agricultural nitrogen input reduction from 233 to 134 TgN yr^−1^ (assuming a global mean current nitrogen use efficiency (NUE) of 50%). Improvements in NUE could increase this critical nitrogen input limit without crossing the nitrogen surplus boundary^10^ (Supplementary Text 5).

### Phosphorus

Phosphorus losses to surface water are largely determined by physical soil erosion rates and the build-up of soil phosphorus content. Annual phosphorus delivery to surface water amounts to 9.7 TgP yr^−1^ (ref. ^35^), which is approximately 40% higher than the globally acceptable phosphorus delivery loss (6.1 TgP yr^−1^) based on critical phosphorus surface water concentrations^36^. Food systems (including agriculture, aquaculture and phosphorus from wastewater; Fig. 3) contribute around 75% to these phosphorus losses^35^ (Supplementary Table 8). Therefore, phosphorus loss from agricultural land should be reduced proportional to its 75% share (that is, from 7.2 to 4.6 TgP yr^−1^ from agricultural soils), reducing both the risk of surface water eutrophication and soil fertility loss.

### Stratospheric ozone

Stratospheric ozone depletion has historically been dominated by the release of chlorofluorocarbons, but since the strict regulation imposed via the Montreal Protocol, the ozone layer has mostly recovered^2^. Currently, N_2_O is the single most important ozone-depleting substance^37^, and food systems are the dominant driver of N_2_O emissions, mainly via fertilizer and manure application. Food systems are responsible for 54–69% (excluding or including biomass burning, respectively) of the anthropogenic N_2_O emissions^38^ (Supplementary Table 9). However, the contribution of N_2_O to stratospheric ozone depletion remains small, and the concentration of stratospheric ozone is expected to return to historical values by the end of this century even without any further N_2_O reductions^39^. We therefore restate the existing boundary for nitrogen surplus (57 TgN yr^−1^), which simultaneously reduces the N_2_O emissions (from 3.9 to 1.8 TgN_2_O-N yr^−1^) associated with exceeding the nitrogen surplus levels^10^.

### Ocean acidification

Ocean acidification has planetary-wide impacts through the loss of marine species dependent on calcium carbonate, and changes to marine carbon storage^40^. Acidification also amplifies threats to marine life deriving from climate change, such as ocean warming and associated thermal stress^41^, and nutrient pollution in coastal zones^42^. Ocean acidification is strongly linked to CO_2_ emissions, one-quarter of which is absorbed by the ocean^43,44^, resulting in lower pH and hence affecting the aragonite saturation state. Food systems now contribute around 25% of the total CO_2_ emissions^12,43,45^ (and a similar rate of cumulative emissions from 1750 from land conversion; Supplementary Text 7), which following the climate change boundary, is set at zero CO_2_ emission by 2050 through limiting emissions from land use change (5.9 GtCO_2_ yr^−1^) and other emissions from pre- and postproduction (4.3 GtCO_2_ yr^−1^) (Supplementary Table 2).

### Aerosol loading

Increasing atmospheric aerosol loading—particular interhemispheric difference in aerosol optical depth (AOD), a measure of aerosol concentration blocking incoming solar radiation^2^—can have critical impacts on atmospheric circulation patterns (that is, monsoon patterns) and the ocean (that is, Atlantic meridional overturning circulation^46^). Overall, anthropogenic aerosol loading is highest in the northern hemisphere^47,48^ where most of the land mass and associated human activities are concentrated, increasing the interhemispheric difference of AOD to 0.076 (ref. ^2^). Sources of aerosols are both natural (dust, sand and wildfire contribute >75% of total PM2.5 mass globally) and anthropogenic (combustion, livestock, energy and industrial production, which contribute ∼25% of PM2.5 mass) but predominant sources can regionally vary^49^.

Food systems are a major contributor to anthropogenic aerosol loading by emitting direct particulate matter (PM2.5; that is, black and organic carbon from biomass burning from land clearance and agricultural waste burning) and secondary particle emissions from fertilizer use and livestock (NH_3_) and energy-related processes in the food supply chain (SO_2_)^50^ (Supplementary Fig. 4). In the northern hemisphere, anthropogenic PM2.5 derives predominantly from fossil fuel combustion^51^ and NH_3_ emissions^49,52^ from animal-based agriculture^50,52–54^. In the southern hemisphere, biomass burning is the main constituent of anthropogenic PM2.5, and is regionally responsible for up to 90% of the PM2.5 concentration^49^. Some estimates suggest that around half of global biomass burning-based PM2.5 (32 Mt yr^−1^) derives from land conversion for agricultural production (14 Mt yr^−1^)^55^. In addition, agricultural waste burning emits another 6.8 Mt yr^−1^. In the southern hemisphere, where land conversion is concentrated (in the tropical forests of Amazon, Congo and Indonesia), the contribution of food systems to PM2.5 concentration may even be higher. Remaining within the PB for aerosol loading requires aerosol emissions to be reduced in both the northern and southern hemisphere simultaneously, to protect local climate functioning, and human health and crop production^46,49,50,54^. We propose that northern hemisphere reductions in PM2.5 concentration can effectively be obtained through reducing NH_3_ emissions in line with the nitrogen surplus boundary (global reduction of NH_3_ emissions from 37 to 20 TgN yr^−1^); while southern hemisphere reductions should be obtained from halting land conversion and associated emissions from biomass burning, in line with the land system change boundary.

### Novel entities

Food systems are responsible for the release of a wide range of novel entities in the environment, such as plastics in food packaging and on-field use^56^; pesticides for crop protection^57^; antimicrobial use in animal husbandry^58,59^; and the introduction of (hybrid) varieties of genetically modified crops. Here, we consider pesticides and antibiotic use as key components of food system-sourced novel entities, considering their wide-spread use for food production and the availability of global data.

#### Pesticides

More than 3 Tg of pesticide active substances (PASs) are used in food production each year^60^ (Supplementary Fig. 6). Around 82% of PASs subsequently degrade into a cascade of compounds; 10% remains as soil residues; 7.2% leaches below the root zone; and 0.1% enters river systems. These residue concentrations in surface water are exceeding safe exposure levels for aquatic biodiversity along 13,000 km of the world’s major rivers^57^; 75% of agricultural land area is at risk of pesticide pollution by at least one active ingredient, while 64% is at risk of more than one active ingredient^61^. Bioaccumulation in organisms, legacy effects and cumulative cocktail loads^62^ pose uncertainties on defining the safe exposure levels for biodiversity, and frustrate the setting of local and global boundaries for pesticide application. We therefore argue for a precautionary approach and propose that the residue concentration of pesticides in the environment should remain within local safe exposure levels for biodiversity, requiring a global reduction in pesticide application of 70% to minimize the area with high pesticide pollution risk, and a reduction of more than 90% to avoid introducing additional pollution risks to the environment (for details see Supplementary Text 9a and Supplementary Fig. 7) compared with the current PAS application rates.

#### Antimicrobial use

Global antimicrobial use for livestock and aquaculture amounts 73–130 kt yr^−1^ (refs. ^58,59,63,64^) and is projected to further increase with the growing demand for animal-sourced foods and the shift towards intensive production. Most of the antimicrobial use is for food animals (73%); around a quarter is for human consumption^64^. Antibiotics accumulate in soils via application of animal manure, increasing antimicrobial resistance risk in soil biota^65^. The subsequent impact on genetic diversity and abundance of soil biota through selection pressure can undermine crucial soil functions that enable biogeochemical cycles of nitrogen and carbon^66^, through which they affect other PB domains. Considering uncertainties regarding the development of antimicrobial resistance across species, and subsequent risks for human and environmental health more broadly^67^, we propose halting prophylactic use of antibiotics in agriculture (that is, preventive use of antimicrobials in healthy animals) in line with World Health Organization recommendations^68^, and halving the overall current average application rates (from 50 to 25 mg kg^−^^1^ of animal) while retaining the productive capacity of livestock systems (Supplementary Text 9b).

## Discussion

Our results show that food systems are the single-largest pressure across Earth system processes and that all proposed FSBs are currently transgressed. It is critical for food systems to operate within a safe space to preserve Earth system stability. Operating outside FSBs is simultaneously putting human health at risk^54^ and undermining the capability for food production itself by polluting the environment^50,61,69^, ultimately increasing the exposure of agricultural land to extreme weather events and driving the loss of biodiversity fundamental for food production (for example, soil microbiomes, and pollinating and pest-regulating organisms). The economic costs of food systems now outweigh benefits, and climate change is expected to further increase economic losses, and to increase food prices and the occurrence of hunger^70^. Without mitigation actions, food systems will contribute up to an additional 0.9 °C of warming by the end of this century^71^.

The FSBs proposed here provide an integrated framework for measuring progress of the performance of global food systems against the PB framework. Since FSBs respond to changes in mitigation cost and many other societal developments, they should be considered dynamic sectoral shares (aligned with the PB framework, albeit fundamentally different from the biophysical thresholds represented by PBs or targets derived from stakeholder negotiation or policy deliberation processes).

This is a first attempt to operationalize the PB framework for food systems across all planetary boundaries; moving towards the safe operating space requires parallel action and transformation across sectors, including moving to carbon-neutral energy and transportation systems.

Our assessment highlights important knowledge gaps: the lack of uniform approaches to defining food systems (that is, what is included and excluded) across different fields of Earth system science, leading to different estimates of the impacts of food systems. Agricultural production data often provide limited granularity for the discard effects of non-food products (for example, biofuel, fibre and timber production), and probably overestimate the impacts of food systems across the PBs. However, available evidence suggests that this component is relatively small (suggesting a mean ∼10% error margin; Fig. 1b). Efforts to ensure internally consistent data collection on the impacts of food systems, following a unified definition and systematic disaggregation of food and non-food agriculture, can make important contributions to addressing these knowledge gaps. Moreover, there is no consistent approach to quantify the FSBs. Some FSBs (that is, climate, and indirectly, ocean acidification and aerosol loading) are partially derived from IAMs^72^, which have been critiqued for their limited capacity to model behavioural systems in relation to the cost-optimization objective, leading to biases towards technological solutions (for example, carbon dioxide removal (CDR)^73^). More ambitious IAM scenarios that assume greater demand-side shifts (for example, dietary changes) may bring emissions closer to zero. However, such behavioural demand-side changes are usually exogenously prescribed in IAMs^74^, implying that models may underestimate demand-side mitigation potential and favour techno-optimist solutions over societal transformation (for example, new technologies for emission efficiencies in meat production, rather than contract-and-converge pathways for per-capita meat consumption). This emphasizes that the 5 GtCO_2_e y^−^^1^ and other FBSs proposed here will be dynamic because there are diverse and emerging combinations of behavioural and technical options to stay within boundaries^75^.

Our integrated framework identifies critical interventions that can tackle multiple issues at once: halting further agricultural expansion is critical to safeguarding biodiversity, sequestering carbon, reducing PM2.5 emissions, maintaining biogeochemical cycles, and retaining blue and green water; restricting nitrogen surplus reduces water and soil pollution, while limiting further ozone depletion and radiative forcing from associated N_2_O emissions^10^; bringing CO_2_ emissions to zero to limit further global warming and further ocean acidification to protect ocean life and aquatic food supply. These interactions underline the cross-cutting nature of food systems, and strongly suggest the need to align mitigation efforts for the food system with existing global governance frameworks, such as under the Convention for Biological Diversity to protect land and biodiversity; and the UN Framework Convention on Climate Change, bringing food front and centre in biodiversity and climate policy.

Existing studies stipulate the potential of food system transformations to bring food systems from the problem to the solution side for the environment, the economy^70^ and human health, through a range of actions including dietary shifts, reductions in food loss and waste, and improved production practices. Further research is urgently needed into how we can transform both the demand and supply side of food systems to move back into the safe space. This research should address how effective policy measures can support such transformative actions, while simultaneously preserving the capacity to produce sufficient, healthy and affordable food for a growing population.

## Methods

### Identifying control variables and present-day food systems’ contributions

The Earth system domains and associated control variables adopted in this study are in principle based on the most recent PB assessment^2^. In addition, we identify recent studies that propose alternative control variables that we consider better adapted to capture food system impacts, or to express the FSB. For biogeochemical flows, we adopted the proposed ‘nitrogen surplus (TgN yr^−1^)’ control variable^10^ rather than the ‘industrial and intentional fixation of nitrogen (TgN yr^−1^)’^2^, and adopt phosphorus loss from soils to surface waters, based on ref. ^36^. For novel entities, we propose two control variables that capture the contributions of food systems to the release of novel entities in the environment (pesticide application and antimicrobial use)^61,64^.

Next, we quantify the present-day contribution of food systems to PB transgressions through a scoping review of recent PB studies and global food systems impacts (Supplementary Table 1). In principle, we consider food systems to comprise both the production part and the processing, distribution and consumption parts (that is, from farm to fork), in line with the ‘agrifood system’ defined by refs. ^76–78^; however, we are sometimes limited by data availability, which implies some elements of the processing part are excluded (Fig. 1a) or not based on recent data (for example, nitrogen surplus estimates are based on 2010 nitrogen data). Although agriculture comprises more than food (also including, for example, crop production for bioenergy and fibres), most production (in terms of mass) is used for human consumption (direct consumption or indirect via fodder production)^4^ which we therefore consider a good proxy for food systems, and comparable to approaches adopted in similar assessments^12^. Equally so, the environmental impacts aligned with the PBs following from agricultural production also appear dominated by food over other uses of agricultural production (Fig. 1b). However, as there is no unified approach to define and quantify the impacts of food systems across the PB domains, the presented numbers in the literature strongly depend on what is included.

### Defining FSBs

We define FSBs as a specific global share of the PB budget. In contrast to PBs, FSBs do not reflect biophysical thresholds; rather, they are a set of science-based shares of food systems consistent with the PB framework. We identified several approaches to define these shares for the food system from the available literature (Supplementary Table 1) that broadly follow three main principles. First, based on the estimated required reduction of food systems only to ensure moving back within the PBs (that is, assuming that the pressure from other sectors remains constant, such as biosphere integrity, freshwater change and novel entities), or assuming proportional reduction across sectors based on current contributions to PB transgressions (for phosphorus, nitrogen, blue water and land use)^5,9^. Second, using top-down economic optimization models to estimate cost-effective mitigation potentials across sectors (that is, for climate change)^72,79^. Third, by considering cross-boundary interactions that provide multiple benefits to various Earth systems (that is, for ozone depletion, aerosol loading and ocean acidification). There is no single framework available to allocate the PB budget within the safe operating space across sectors, and no approach is without limitations: assuming proportional mitigation across sectors may not represent the most cost-effective target, while mitigation estimates from top-down economic optimization models can be dependent on a selection of mitigation options considered and on the assumed carbon price (that is, for the climate change FSB). This means that FSBs also can be adjusted over time based on new insights on reduction potentials per sector. Detailed approaches for defining the FSB are provided in the Supplementary Information for each Earth system domain. Alternative methods used in the literature to define FSBs are based on mitigation potentials, such as the mitigation options from dietary change^54^, or on bottom-up approaches that identify per-capita budgets and aggregate those to the global level to derive a global estimate of minimum requirements (as adopted in ref. ^80^, which adopted a human-rights approach to define Earth system boundaries). Such approaches depend strongly on the assumptions made to derive per-capita budgets (including population trajectories), and on subsequent aggregation methods. For example, per-capita GHG emissions depend largely on the composition of food intake and can vary regionally depending on where the food is sourced.

### Reporting summary

Further information on research design is available in the Nature Portfolio Reporting Summary linked to this article.

## Supplementary information

Supplementary Information Supplementary Texts 1–10, Figs. 1–8 and Tables 2–12.

Reporting Summary

Supplementary Table 1 This Excel file (incl. the sheets as further described below) contains the references included in the review in support of Table 1 and Fig. 2 in the manuscript. It includes primary references that support underlying data for estimating the current status of food systems across the PBs (see Sheet 1. Current state - primary references), as well as secondary references (see Sheet 2. Current state - secondary references) that are used and referred to in primary references, indicated in bold on Sheet 1. It also includes references used for identifying the food system boundary (see Sheet 3. Boundary state).

## Data Availability

The data used for the generation of the food system status presented in Fig. 2 and Table 1 were obtained from the literature (Supplementary Table 1). The data needed to reproduce Figs. 1, 3 and 4 and Supplementary Figs. 1–6 are available via Zenodo at 10.5281/zenodo.17397894 (ref. ^81^).

## Code availability

The code needed to reproduce Figs. 1, 3 and 4 and Supplementary Figs. 1–6 is available via Zenodo at 10.5281/zenodo.17397894 (ref. ^81^).
