# Supplementary material for: Identifying the safe operating space for food systems
Source: Nat Food. 2025 Oct 31;6(12):1153–63. doi: 10.1038/s43016-025-01252-6 (PMC12717010; doi:10.1038/s43016-025-01252-6)
Supplement: Supplementary file 1 — Supplementary Texts 1–10, Figs. 1–8 and Tables 2–12. [file 43016_2025_1252_MOESM1_ESM.pdf]

nature food

Supplementary information

https://doi.org/10.1038/s43016-025-01252-6

Identifying the safe operating space for food systems

In the format provided by the authors and unedited

Supplementary Information

Supplementary Text 1. Climate change

Supplementary Table 2 Emissions from agriculture, forestry, and land use. CO_2 equivalents (CO_2e) are estimated for methane (CH_4) and nitrous oxide (N_2O) emissions based on IPCC GWP100 conversion factor. Source of data: [1] for 1a-c and [2] for 2.

Source of emissions	GHG	Mean annual net flux	% of net global emissions	Range (Gt CO_2e yr^{-1})	Time range
**1. Agriculture, forestry and land use (AFOLU)**	CO_2, CH_4, N_2O	11.9 Gt CO_2e yr^{-1}	21%	± 4.4	2010-19
**1 a. Land use change**	CO_2	5.9 Gt CO_2e yr^{-1}	11%	± 4.1	2010-19
**1 b. Direct methane emissions from agriculture**	CH_4	157 Mt yr^{-1} which is 4.2 Gt CO_2e yr^{-1}	8%	± 1.3	2010-19
**1 c. Direct nitrous oxide emissions from agriculture**	N_2O	6.6 Mt yr^{-1} which is 1.8 Gt CO_2e yr^{-1}	3%	± 1.1	2010-19
**2. Other food system emissions (pre- and post-agriculture)**	CO_2, CH_4, N_2O	5.8 Gt CO_2e yr^{-1}	10%	-	2018
**Total emissions attributable to agriculture, land use and food systems**	CO_2, CH_4, N_2O	**17.7 Gt CO_2e yr^{-1}**	**31%**	-	**2010-2019; 2018**

Supplementary Text 1a. CO_2 equivalent emissions

Figures on net emissions from food systems depend strongly on what is included. The IPCC applies an accounting system that treats agriculture, forestry and land use (AFOLU) as a sector and assigns emissions associated with other aspects of the food supply chain – including energy use on-farm and post-farmgate, transport, cold storage, processing, retail, catering, food management in the home, and waste – to other sectors. Here, we use the AFOLU emissions provided in [1], and pre-and post-agriculture emissions (from food transport, waste disposal and supply chains, including energy use for fertilizer production) estimated in [2] to estimate the present-day contribution of food systems to annual CO_2-equivalent emissions (**Supplementary Table 2)**. In contrast to the control variable (atmospheric CO_2 concentration) used in the planetary boundary framework [3], we use emission levels expressed in CO_2 equivalents, including non-CO_2 gasses (CH_4 and N_2O). The majority of methane emissions (60%) derive from enteric fermentation in livestock, while 25% comes from rice cultivation, and smaller portions from manure management and biomass burning [1]. Nitrous oxide emissions derive predominately from agricultural soils (related to application of manure and fertilizers) [4]. This suggest food systems are in total responsible for 17.7Gt CO_2e yr^{-1}. Estimates based

on life cycle assessments [5] that include only food items (and exclude non-food agricultural production, such as fibers and biofuels) provide similar quantifications (16 Gt CO_2e yr^{-1}).

The food system boundary is based on a range of studies using both top-down sector optimization models (IAMs) to allocate the remaining carbon budget specified within RCP pathways based on a variety of mitigation scenarios, and bottom-up approaches that aggregate the mitigation potentials of candidate technologies (**Supplementary Table 3**). All studies in **Supplementary Table 3** point to a residual ±5 GtCO_2e yr^{-1} emissions from agriculture in 2050, consistent with staying within the carbon budget and planetary boundary. These residual emissions include methane and nitrous oxide (mostly from livestock production), which have a higher global warming potential than CO_2, but shorter half-life. The FSB reduces carbon dioxide emissions to zero through halting land use change (aligned with the Land Use Change Boundary), achieving full decarbonization of energy use in the food supply chain, and stopping the burning of agricultural lands. These constitute a highly ambitious set of actions but are consistent with other boundaries and previously proposed decarbonization pathways [6]. Mitigation scenarios that assume more ambitious demand-side changes (e.g. lifestyle changes with lower energy use and dietary shifts) could further reduce residual emissions from agriculture (particularly CH_4 and N_2O from livestock from less meat-intensive diets) [7]**.** Cost-effective agricultural mitigation estimates from IAMs are generally much larger compared to (bottom-up) sectoral estimates, amongst others because baseline emissions of non-CO2 gasses are often assumed higher in IAMs [8]. However they are limited as they only consider a selected group of mitigation measures (i.e. mostly supply-side, see [9] and **Supplementary Table 3**).

We have now surpassed our ability to stay below 1.5 °C through emissions reductions alone; removals of emissions – also known as carbon dioxide removal (CDR) or negative emissions – are now a universal component of credible pathways to achieve 1.5 or 2 °C, particularly from the land sector. Illustrative Mitigation Pathways (IMPs) show that all mitigation scenarios include negative CO_2 emissions from the land sector, as well as negative emissions from BECCS [1]. Agricultural land can remove greenhouse gases from the atmosphere via carbon sequestration in croplands and pastures. There is considerable technical potential to increase removals of carbon dioxide into agricultural systems, both below ground in soils, and above ground into perennial crops, agroforestry and buffer strips [10–12]. Below ground, the potential for soil carbon sequestration on remaining agricultural lands is estimated at 1-4 Gt CO_2 eq yr^{-1} [13]. Estimates at the lower side of the range take limitations to SOC sequestration in account in extensively used grazing land, from nutrient (N) availability requirements, and availability of organic fertilizer [13]. Both above and below ground carbon sequestration potential from soil

carbon management in croplands, grasslands, agroforestry, and biochar is estimated around 3.4 (1.5–5.5) Gt CO_2 eq yr^{-1} (at a carbon price of USD 100 t CO_2 eq^{-1}), with a technical potential of 9.5 (1.1–25.3) Gt CO_2 eq yr^{-1} [1] based on Roe et al. (2019). It is important to note that carbon stocks reach saturation points which implies the rate of C sequestration slows down over time. Furthermore, decomposition of organic material will return carbon stocks to the atmosphere.

Supplementary Table 3 Approaches to estimate the emissions reduction potential in agriculture for setting the food system boundary for climate change. Residual emissions under Results are based on baseline emissions for 2050 (7.1 - 8.0 $GtCO_2$ yr_{-1}), estimated by [7]. Mitigation potential estimates under Results are based on carbon price USD 100, which is the 2020 carbon price compatible with 1.5 °C [15].

Approach	Study	Results	Notes and assumptions
Integrated Assessment Model (IAM): Optimized allocation of emissions by sub-sector on RCP2.6 decarbonisation pathway compatible with Paris agreement	[16]	Residual agricultural emissions of **4.7 - 5.3 Gt CO_2 eq yr^{-1}** in 2050	Allocation of remaining agricultural CH_4 and N_2O emissions reductions by 3 IAMs that do not include dietary change.
IAM: Optimized allocation of emissions by sector or sub-sector	[7]	Agricultural emissions reduction potential of 2.6-3.3 Gt CO_2eq yr^{-1} at USD 100 carbon price, equating to residual emissions of **3.8-5.4 Gt CO_2eq yr^{-1}** in 2050	Allocation of agricultural CH_4 and NO_2 emissions reductions by 4 IAMs including dietary change. The 4 IAMs project 2050 baseline emissions for non-CO2 agricultural gases of 7.1-8.0 Gt CO_2 eq yr^{-1}
Sectoral: Bottom-up sum of technical and/or economic mitigation potentials across practices	[11]<br>[17]<br>[18]	Agricultural emissions reduction potential of 0.3-2 Gt CO_2eq yr^{-1} at USD 100 carbon price, equating to residual of **5.1-7.7 Gt CO_2eq yr^{-1}** in 2050	Range across studies calculated by [7].
Both IAM and sectoral	(Roe et al., 2021)	IAM: Agricultural emissions reduction potential of 0.3-5.5 Gt CO_2eq yr^{-1} at USD 100 carbon price equating to residual of **1.6-7.7 Gt CO_2eq yr^{-1}** in 2050<br><br>Sectoral: Agricultural emissions reduction potential of 0.6-0.7 Gt CO_2eq yr^{-1} at USD 100 carbon price, equating to residual of **6.4-7.4 Gt CO_2eq yr^{-1}** in 2050	Allocation of agricultural CH_4 and NO_2 emissions reductions by 6 IAMs. The wide range of results reflects differences in assumptions among the IAM included scenarios (n=131) and level of inclusion of various practices (i.e. CDR).<br><br>More conservative assumptions on scale of adoption of practices than in IAMs. By including carbon removals (sequestration) on agricultural lands, net agricultural emissions drop to 0.7-3.9 Gt CO_2eq yr^{-1} in 2050.
Synthesized IAM and sectoral analysis	IPCC AR6 [1]	Agricultural emissions reduction potential of 0.3-1.3 Gt CO_2eq yr^{-1} at USD 100 carbon price, equating to residual of **5.8-7.7 Gt CO_2eq yr^{-1}** in 2050	IAM assessment derived from AR6 database and sectoral assessment from Roe et al 2019 (an earlier iteration of Roe et al 2021). Dietary change not included. By including carbon removals (sequestration) on agricultural lands, net agricultural

	emissions drop to 3.8-7.3 Gt CO_2eq yr^{-1} in 2050.

Supplementary Text 1b. Radiative forcing

Radiative forcing has increased to +2.79 (1.78 – 3.61) W m^{-2} in 2023, compared to 1750, mostly from increased GHG concentrations and a reduction in the magnitude of aerosol forcing. This is slightly lower than the previous year when radiative forcing amounted +2.91 (2.19 - 3.63) W m^{-2} in 2022, due to exceptionally high aerosol emissions from biomass burning in 2023 [19]. The planetary boundary has been defined at a 350 ppm CO_2 concentration, corresponding to a forcing of around 1.2 W m^{-2}, given the variation during the Holocene while the upper end of the zone of increasing risk has been set at 1.5 W m^{-2} [3]. This means that we are beyond the safe operating space for climate. For comparison, remaining within temperature increase of 1.5 °C of warming suggest a 1.9 W m^{-2} boundary [20]. The first PB assessment in 2009 [21], mostly focused on the atmospheric concentrations of CO_2, but since then it has become increasingly clear that changing concentrations of non-CO_2 gasses (positive forcing effect) and aerosol loading (negative forcing effect) can strongly change forcing (in contrast to global non-CO_2 GHG and aerosols concentrations canceling each other out). This means that the climate change PB can be transgressed in terms of radiative forcing, while being below a concentration of 350 ppm CO_2, and emphasizes the importance of accounting for non-CO_2 gasses in the assessment of the climate change PB (**Table 1**).

Effective radiative forcing estimates are based on a range of forcers (both negative and positive radiative forcing). The five main elements contributing to positive forcing (CO_2, CH_4, N_2O, Ozone and halogenated GHG) have contributed around + 3.92 W m^{-2}, while negative forcers (aerosols and land use change) have contributed –1.18 W^{-2} since 1750 up to 2022 [22]. **Supplementary Table 4** shows the contributions of different GHG's and other forcers to the change in radiative forcing, and the subsequent contribution of food systems to the emissions of these forcers based on its atmospheric lifetime. This suggests that food systems have been responsible for at least +0.98 W m^{-2} of the positive radiative forcing (excluding halogenated GHGs, ozone and water vapor), and -0.29 W m^{-2} of the negative radiative forcing (assuming all land use changes are food related), suggesting a net increase of around +0.69 W m^{-2} (24% of net increase in forcing). The positive forcing (+0.98 W m^{-2}) is likely on the lower end of the range as food systems also contribute to the release of tropospheric ozone [23] which is not accounted for in our balance (**Supplementary Table 4**). Despite atmospheric lifetimes being >100 years for both N_2O and CO_2, we are limited by data to (reliably) attribute cumulative N_2O and CO_2 emissions that extend back more than 1920 (for CO_2) and 1970 (for N_2O) [24]. For the negative forcers, we assume that at least three-quarters of the aerosols-radiation and

aerosol-cloud interactions (approximated by particulate matter concentrations, PM2.5) are of natural origin (**Supplementary Text 8**). From the remaining anthropogenic aerosols, exact contributions from the food system are uncertain [23], although it can be as high as 58% of the primary PM2.5 emissions [25]; and over >80% of precursors (e.g. NH_3) forming secondary PM2.5. There are also uncertainties regarding the exact contribution of primary and secondary PM2.5 emissions to total concentrations, due to highly variable contribution from precursors. It Is therefore challenging to attribute the food system share of negative forcing from aerosol-interactions (**Supplementary Text 8**). Therefore, we use the middle (mean) of the range provided by [25] and [23] for primary PM2.5 concentrations (43%) from anthropogenic PM2.5, and ignore the variable contribution from precursors (NH_3). This might underestimate the actual contribution of food systems to negative forcing.

Supplementary Table 4. Forcers and relative EFR contribution over the full period (1750-2022) as presented in [22]. Food system contributions are based on more recent cumulative emission estimates approximated by their average atmospheric lifetimes (limited by historical data availability for N_2O) [1,23,26]. For aerosol interactions (here based on $PM_{2.5}$ concentrations), we assume that natural sources of aerosol loading have remained constant over time, hence have not contributed to the increase in EFR from aerosol-radiation and aerosol-cloud interactions. We estimate that food systems contribute around 43% (median of range) to anthropogenic $PM_{2.5}$ (which make up 25% of the total PM2.5, see [27] concentrations, and that $PM_{2.5}$ concentrations are direct proxy for both aerosol radiation interaction, and cloud interactions. Global estimates of the total anthropogenic $PM_{2.5}$ concentrations attributable from food systems (from primary and secondary components) are uncertain. Method adopted by [22] to calculate EFR is based on AR6 WGI.

Forcers	EFR contribution (1750-2022) (W m^{-2})	Food system contribution	Approach	Reference
**CO_2**	2.25	0.56 (25%)	Share based on cumulative CO_2 emissions from land use change (147 GtC) and total cumulative anthropogenic emissions (584 GtC) (lifetime 100 year: 1920–2020).	[24]
**CH_4**	0.56	0.27 (48%)	Share based on cumulative CH_4 emissions from agriculture and total cumulative anthropogenic emissions (lifetime 15 years: 2009–2023)	[28] EDGAR v.06, CH_4 emissions per sector (Agriculture)
**N_2O**	0.22	0.15 (68%)	Cumulative N_2O emissions from agriculture and total cumulative anthropogenic emissions (lifetime >50 years, 1970–2023)	(Crippa et al., 2024;) EDGAR v.06, N_2O emissions by sector (Agriculture)
**Halogenated GHGs**	0.41	n.a.	n.a.	n.a.
**Ozone**	0.48	n.a.	n.a.	n.a.
**Stratospheric water vapor**	0.05	n.a.	n.a.	n.a
**Aerosol-radiation interaction**	-0.21	-0.02 (11%)	43% (±28–58%) of anthropogenic $PM_{2.5}$ (25% of total $PM_{2.5}$), total contribution of 11%.	[23,25,27]

**Aerosol-cloud interactions**	-0.77	-0.08 (11%)	Idem	idem
**Land use**	-0.2	-0.19 (98.5%)		[29]
**Light absorbing particles on snow and ice**	0.06	n.a.	-	n.a.
**Aviation induced cirrus**	0.05	n.a	-	n.a.
**Positive radiative forcing from food systems**		+ 0.98 (34%)	Excluding halogenated GHGs, Ozone, water vapor, absorbing particles on snow/ice, and aviation induced cirrus	
**Net radiative forcing from food systems**	+2.91	+0.69 (24%)	Excluding halogenated GHGs, Ozone, water vapor, absorbing particles on snow/ice, and aviation induced cirrus	

Supplementary Text 2. Land system change

Supplementary Text 2a. Global intactness

To estimate the present-day contribution of the food system to the changes in intactness, we use global estimates on land use cover from [30]. These suggest that in 2022, agricultural land covered ±48 Mkm^2, of which cropland covered 15.7 Mkm^2 and grazing lands (permanent meadows and pastures) covered 32.1 Mkm^2. The global agricultural land area covers more than one-third of the total land area (130 Mkm^2) [30]. Estimates of agricultural land area vary based on their approach and definitions. Other (recent) estimates from i.e. [31] suggest croplands cover around 12 Mkm^2; while [32] suggest that cropland area ranges between 11-19 Mkm^2. To be consistent with i.e. IPCC and the SDG framework, we adopt the FAOSTAT approach and data for the estimate of global cropland extent. Most assessments agree that approximately 50% of the world's terrestrial land area remains intact [33]. We define intactness as the state of an ecosystem's species composition and abundance being largely unimpaired from post-industrial human alteration [34]. [33] propose an intact area boundary of 50-60% of the Earth land surface to reduce extinction risk and maintain critical Earth system functions. The extent of intactness required converges around 50% independently of most of these definitions. Intact lands are distinct from protected areas. Intactness refers to the intactness of the ecological community (species composition) and biosphere function; protection refers to a legal designation whose aim may often, but not always include maintaining, or restoring intactness. Halting species loss requires distributing this intactness across unique ecoregions, whereas retaining climate mitigation potentials requires focused intervention on carbon dense ecosystems, notably 50%, 85%, and 85% retention of temperate, boreal, and tropical forests respectively [3], as adopted in PB3.0 (Text S2b).

Currently, around 45-50% of the land cover is considered natural, or intact [33,34]. Therefore, we restate that no new conversion of natural to agricultural land area for food production should take place (i.e. zero conversion of intact nature) to preserve the remaining intactness globally. This implies that projected increases in crop production should derive from existing agricultural areas. Respecting this boundary while increasing productivity on existing lands (in combination with a dietary shift) can produce sufficient food for feeding 10 billion people in 2050 [35,36].

Supplementary Text 2b. Loss of forest biomes

Forest biome boundaries are defined based on Richardson et al. (2023) similar to the approach adopted in Steffen et al. (2015). The (regional) planetary boundaries for temperate (>50%), tropical

(>85%) and boreal (>85%) forests (**Table 1**) are based on the potential of the forest cover to influence global climate; and the potential of self-reinforcing feedbacks regulated by land cover change that could further induce forest cover loss. Globally, forest loss is predominantly driven by agriculture (51%, including shifting cultivation) [38], with even higher shares in the tropics (90-99% of forest loss is agriculture induced), mainly for pasture, and oil crops and soy production used primarily for animal feed [39]. The land conversion rate of natural land to crop- and rangelands is distributed unequally, with the largest rate in intactness loss in Africa and South-East Asia [31]. Despite regionally high expansion rates, the global agricultural land area used has been relatively stable over the past two decades [30], mostly due to forest increase on former agricultural lands in Europe. Overall, cropland increased with 0.8 Mkm^2 while pastures reduced with 1.6 Mkm^2 since 2001 [30]. Around half of the cropland expansion came at the costs of natural vegetation; the other half represents pasture conversion and re-cultivation on abandoned land [31].

The distribution of intact forest in the three forest biomes is unequally distributed (**Supplementary Fig. 1**), with boreal forest remaining largely intact while tropical and temperate forest are far below intactness boundaries. Here we have evaluated the extent of crop and rangeland distribution, as well as forest intactness across the three forest biomes (tropical, temperature and boreal forests), based on the sub-biomes represented in [40]. We use intactness data from an ensemble dataset of land where natural processes predominate (LNPP), presented in [33,34] further described in [41]. Crop and grazing land extent derive from HYDE V3.2.1 [29].

We find remaining intactness of tropical forest biomes (8 Mkm^2, 34%), temperate forest biomes (4.7 Mkm^2, 24%) and boreal forest biomes (13.5 Mkm^2, 92%). These deviate from the remaining intact forest estimates in [3] provided in **Table 1**, which include a range of *continental* estimates of remaining forest cover for each biome. Proportionally reducing the total forest deficiency (**Supplementary Figure 1**, red striped bars, summed for tropical forest: 12.2 Mkm^2, and temperate forest: 5.4 Mkm^2) back into the planetary boundaries for both agriculture and other land uses, suggests that agricultural land should reduce with 3 Mkm^2 in temperature forest biomes, and 5.5 Mkm^2 in tropical forest biomes, assuming other land uses would proportionally reduce.

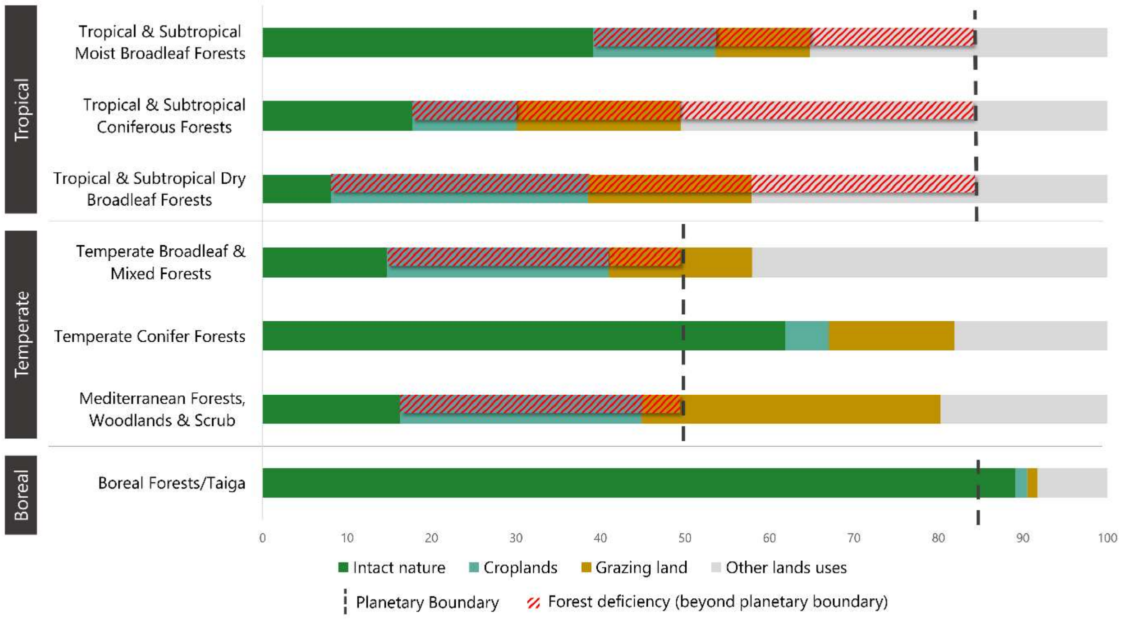

Supplementary Figure 1 Distribution of intact nature, croplands, grazing land (including extensive rangelands and permanent pasture) and other land uses, across boreal, temperate and tropical forest biomes relative to their total biome size (in %). We include both intensive and extensive rangelands here as both generally come at the costs of forest ecosystems. Distribution per (sub-)biome was derived from the estimated area of intact nature [33,41], agricultural land [29] and remaining other land uses at ecoregion level [40].

Supplementary Text 2c. Ecoregion assessment of intactness

We extend the proposed 50% global intactness boundary to ecoregions and suggest that at least 50% of intactness should be preserved to protect ecological, hydrological and climatic functions in each ecoregion [33]. Currently, 69% of the ecoregions have less than 50% intact area; and 45% of ecoregions (or 27% of land surface) have less than 10% intact area [34,42]. Retaining 50% intactness across ecoregions requires regional restoration efforts that would sum up to 23.9 Mkm^2 of restored intactness [34] . The greatest deficits are in the tropical and subtropical dry broadleaf forests (0.8% intact), tropical and subtropical coniferous forests (1.0% intact), flooded grasslands and savannas (6.9% intact) and Mediterranean forests, woodlands and scrub (7.3% intact). All grasslands, except for montane grasslands, are less than 12% intact.

We propose that agricultural land should be limited to cover <50% of the ecoregion area (zone of increasing risk), representing the upper end of the range to also allow for other land uses i.e. human settlement, infrastructure, preferably 40%. We use intactness data presented in [33] and derived from [41] at 1 km^2 spatial resolution, described under 'Loss of forest biomes' above. For agricultural land, we use cropland and grazing land (considered 'permanent pasture' under FAO's definition after 1960, including extensive rangelands and permanent pasture) data from HYDE V3.2.1. [29]. We subsequently

calculate the land area (%) covered by intact nature, cropland and grazing land of each ecoregion [40]. We find that in 10% of the ecoregions, cropland alone is moving in the zone of increasing risk (>40% coverage) with 5% of the ecoregions transgressed (>50% coverage) from cropland alone. Using *all agriculture* (including both cropland and intensive and extensive grazing land) in each ecoregion (**Supplementary Figure 2**), we find that in 43% is moving in the zone of increasing risk of 40% (**Supplementary Figure 2d**), and 34% crossing the boundary (>50%). When excluding extensive grazing land, as it generally has lower impact on ecosystem functioning and can support (close to) intact grasslands ecosystems, **~**20% rather than 34% of the ecoregions are transgressed (from cropland and permanent pasture), but there are major uncertainties regarding the level of intactness in grazing lands.

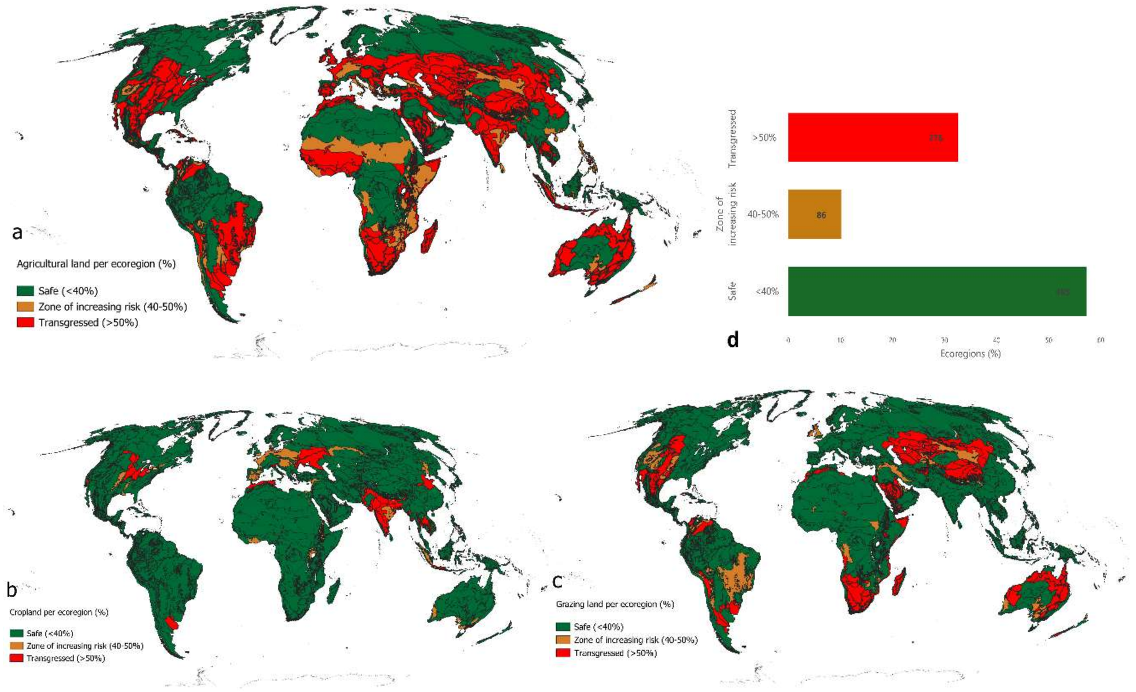

Supplementary Figure 2 a) Agricultural land area per ecoregion (%), comprising cropland (arable land and permanent crops) (**b**) and grazing land (permanent pasture and extensive grazing lands (**c**). Number of ecoregions that area transgressed from agriculture (including extensive rangelands). (**d**) Numbers in bars indicate the number of ecoregions within each category. Green ecoregions have agricultural, crop- or grazing land covering < 40% of the total land area within the ecoregion. Orange regions are in the zone of increasing risk between 40-50% of agricultural, crop-, or grazing land area, while red regions indicate transgression of the intactness boundary from agricultural, crop-, or grazing land (>50% of the land area) alone. Data derives from [29,40]

Supplementary Text 3. Biosphere Integrity

Supplementary Text 3a. HANPP

Planetary boundary and control variable

HANPP has a long history of analysis although it was first used in the planetary boundary context in PB3.0, replacing Biosphere Integrity Index (BII) by making the case that the link between BII and Earth System Functions is poorly understood [3]. Terrestrial HANPP is a proxy of photosynthetic energy and materials flows into the biosphere and thus its justification as a control variable for biosphere integrity [3,43,44]. HANPP sets a limit to human appropriation of biosphere NPP (or HANPP) measured as a fraction of Holocene NPP, to avoid human society from substantially compromising the energy flow to the biosphere [3]. HANPP can then be estimated both as a fraction of potential natural NPP – 15.7% in 1950 and 23.5% in 2020 [45]– and the Holocene mean NPP – 25-30% today [3,43,44]. The PB for HANPP should be set in relation to preindustrial Holocene mean NPP (55 GtC yr^{-1}), and not the current CO_2-enriched potential (70 GtC yr^{-1}), because this enrichment represents a resilience response that dampens the magnitude of Anthropocene warming [3]. Holocene NPP of the terrestrial biosphere has been relatively stable at 55.9 GtC yr^{-1}, varying around 1.1 GtC [3].

In line with Richardson et al. (2023), we define HANPP as the actual NPP harvested (NPP_{harv}), plus the potential NPP inhibited from anthropogenic land use changes (NPP_{luc}) compared to an undisturbed reference situation. There are observed relationships between HANNP and species loss where levels of 20-30% HANPP are consistent with estimates of global species endangerment and loss [3]. A provisional boundary is proposed for 10% HANPP of preindustrial Holocene mean NPP (i.e. 5.5 GtC yr^{-1} of HANPP) with the zone of high risk set at 20%. This implies that >90% of the Holocene mean NPP plus the resilience response (> 64.5 GtC yr^{-1} in total) should remain intact.

Present-day contribution of food systems

Cropland and human settlements have the highest value of HANPP (70-85% of potential NPP). The present-day human appropriated NPP (hereafter 'HANPP') from inhibited NPP (NPP_{luc}) and extracted NPP (NPP_{harv}) amounts 13-16.8 GtC yr^{-1} (20-30% of total NPP) **(Supplementary Table 5**). NPP_{harv} comprises crops (2.08 Gt C yr^{-1}), residues (1.6 Gt C yr^{-1}), wood and timber (1.36 and 0.66 Gt C yr^{-1}, respectively), and grasslands (0.65 Gt C yr^{-1}) [44]. In total, NPP_{luc} (5.6-7.4 Gt C yr^{-1}) and NPP_{harv} from crops, residues and grasslands (4.3 Gt C yr^{-1}, excluding wood and timber) are attributable to agriculture and amount 9.9—11.7 Gt C yr^{-1}, which is 72—85% of the current HANPP, assuming all NPP_{luc} is attributable to agriculture. For NPP_{harv}, including non-food crops such as biofuels and fibres,

[46] shows that the majority (90%) of the blue and green water use (evapotranspiration) related to crops—which is closely related to NPP—is used for food. We therefore can assume that an equal proportion of agricultural HANPP is used to produce food, rather than other crop uses, such as biofuel and fibre production.

In regions where land-use intensification techniques boost plant productivity, particularly on croplands in industrialized agriculture or in arid areas with high irrigation potential, NPP_{luc} can be higher compared to Holocene values, leading to negative values for HANPP if harvested biomass does not exceed the human-induced increase in NPP.

Bringing food systems back within planetary boundaries and preserving NPP for biosphere requires increasing productivity on existing agricultural land (increasing overall NPP) as well as reducing food system energy inefficiencies by reducing food loss and waste, and the use of crops for animal fodder, in order to limit required agricultural land and preserve a higher share for natural processes.

Supplementary Table 5: Global HANPP estimates (in GtC yr^{-1} and %) from various sources [3,43,44].

	2000	2016	2020
	Haberl et al. (2007)	Stenzel et al., (2024)	Richardson et al. (2023)
1. NPP_{harv}	8.18 (53%)	6.37 (46%)	11.2 (67%)
1a. Crops	-	*2.08*	-
1b. Residues	-	*1.6*	-
1c. Wood and timber	-	*1.36 and 0.66*	-
1d. Grasslands	-	*0.65*	-
2. NPP_{luc}	6.29 (40%)	7.45 (54%)	5.6 (33%)
3. Others (human induced fires)	1.14 (7%)	-	-
Total global HANPP	15.6	13.8	16.8

Supplementary Text 3b. Ecosystem integrity

Planetary boundary and control variable

Biosphere functional integrity is the state of an ecosystem's capacity to provide contributions of Earth system or ecosystem processes, independently of degree of modification or species composition. It is measured as natural or semi-natural habitat within a 1 km radius, with minimum area and quality characteristics determined by provisioning of multiple ecosystem services including those necessary to food production [33]. Such embedded habitat is equally important for maintaining connectivity and gene flow and has been demonstrated at local scales although no global assessment exists as of yet. Biodiversity loss in agricultural lands is strongly driven by intensification and habitat fragmentation, including the loss of conservations structures in agriculture. Recent assessments find that pollination, pest regulation, pollution and sediment retention in agricultural and urban landscapes are compromised below 20-25% habitat per km^2 and are generalizable lost <10% habitat per km^2 [47]. Current assessments also find that between 33% [41] and 66% of agricultural lands fall below the 20-25% threshold and therefore have insufficient ecosystem integrity to support ecosystem service provisioning, notably in agricultural and urban lands which have strong dependencies on pollination, pest regulation, and capture of nutrient and novel entity pollution [34,47,49]

Ecosystem functional integrity was added by [33] in the Earth Commission to capture the ecosystem's capacity to provide contributions of Earth system or ecosystem processes. The safe and just boundary is set here at 20-25%. It was also included in the EAT-Lancet Commission [16], emphasizing that staying within boundaries also required fine-scale conservation efforts on already converted

lands (in contrast to remaining within the boundary for intact lands). They adopted a less stringent boundary of 10% of ecologically conserved land per km^2. Based on the above, we adopt the proposed minimum of 20-25% ecologically conserved land at smaller scales (< $1km^2$). This should be integrated into agricultural systems to safeguard habitat connectivity and local ecosystem functions, which is essential to protect biodiversity and support food production. Restoring functional integrity can be achieved by embedding natural habitat in agricultural lands such as agroforests, hedgerows, no-mow zones or riparian buffers.

Supplementary Text 4. Freshwater change

Planetary boundary definition

The initial planetary boundaries framework [21] includes a global boundary for human freshwater use with the control variable "total human consumptive water use". It adopts 4,000 $km^3\ yr^{-1}$ as the freshwater boundary, based on volumes of inaccessible flows and ecosystem flow requirements subtracted from total global runoff, suggesting the boundary is not yet transgressed (**Supplementary Table 7**). Subsequent bottom-up studies have downscaled specific environmental flow requirements (EFR) to river basin level, indicating a stricter boundary of 2,800 $km^3\ yr^{-1}$, but with a large uncertainty range (1,100 – 4,500 $km^3\ yr^{-1}$) following different approaches to calculate EFRs [50]. Additional studies suggests that *regional* EFR boundaries are crossed already in many river stretches, particularly in Southern Europe and Asia, and the Western part of US [37,51].

Recent updates to the planetary boundary for freshwater include a control variable for both blue and green water, and adopt a local (gridded) approach accounting for both water deficits (dry events) and excesses (wet events) [52]. More specifically, local variability envelopes (5th-95th percentile) of stream flow (blue water) and soil moisture (green water) are defined from the pre-industrial baseline period. Subsequently, monthly deviations from these variability envelopes are aggregated to the global ice-free land area (%) that is beyond local variability envelopes. This articulation of the freshwater change boundary thus captures a change in the global frequency and extent of dry and wet events for both blue and green water compared to the pre-industrial period [3,52]. It suggests that currently 18.2% (blue water) and 15.8% (green water) of the global land area is beyond local variability envelopes, which is beyond the area typically experiencing such transgressions under pre-industrial conditions (10.2% for blue water, 11.1% for green water, respectively) [3,52].

Based on the latest PB science, we adopt the two-tailed freshwater change approach for green water. Due to the limited evidence available to express the present-day contribution of food systems' to the changing frequency of freshwater availability, it is also challenging to define a FSB using the new freshwater PB control variables. We therefore express the blue water boundary for food systems on the existing boundary based on local EFR, which is better established across the scientific literature [50,51,53]. For green water, we adopt a preliminary approach based on the new freshwater change PB that we further describe below.

Present-day contributions of food systems

Agriculture is the largest water user in terms of blue water withdrawals and consumptive use. Estimates of irrigation withdrawal and consumptive use amount to around 2,400—2,700 $km^3 yr^{-1}$ and 1,200—1,800 $km^3 yr^{-1}$, respectively (of which around 545 km^3 of consumptive use derives from groundwater) (**Supplementary Table 6**). Consumptive use estimates are lower than withdrawal estimates, as a part of withdrawals returns to the river system and can potentially be appropriated elsewhere. Irrigated agricultural area has rapidly expanded and now covers 3.1—3.4 Mkm^2 which is around 20% of the total cropland, while supporting 40% of the global food production [54]. Agriculture's consumptive use estimates range between 1,200—1,800 $km^3 yr^{-1}$. Estimates at the lower end of the range generally seem to derive from hydrological dynamic models simulating irrigation consumptive use, while the upper end of the range represents crop water footprint analyses (i.e. [36] **(Supplementary Table 6).** Studies suggest that the agricultural share of the global freshwater consumptive use is 84% [55], based on the (rather high) estimate of total consumptive use by [56] (**Supplementary Table 6**). More recent water consumption estimates derived from hydrological model ensembles (i.e. [54] suggest a lower share of consumptive use attributable to agriculture (around 70%). Crop water footprint assessments (i.e. [36], however, appear to confirm agriculture's high share of consumptive use estimates. Considering the large range of estimates and therefore uncertainty of agriculture's contribution (%) to freshwater consumption, we use the most recent ensemble-based study from [54] in Table 1, although a full overview of the references considered is provided in **Supplementary Table 6**.

Groundwater consumptive use for irrigation is estimated at 545 $km^3 yr^{-1}$ [54]. Around 70% of the total pumped groundwater is used for irrigation purposes [57]. Groundwater extraction is exceeding local recharge levels in many regions, particularly in intensively irrigated regions leading to dropping groundwater levels and affecting ground-water dependent ecosystem [33]. Strong links between ground and surface water imply that groundwater withdrawals are breaching critical streamflow levels (based on 90th percentile over 5 years) through connected water systems in 15-21% of the watersheds [57]. Hence, groundwater recharge volumes should be used to locally guide the amount of groundwater that can safely be extracted to prevent crossing of EFRs [58].

When including *green* water consumptive use (5,700-7,500 $km^3 yr^{-1}$ for cropland and 1,700-2,900 $km^3 yr^{-1}$ for grazing lands), the total consumptive water use of agriculture increases to around 8,400-14,300 $km^3 yr^{-1}$ (**Supplementary Table 6**), of which >80-90% is used for food purposes (10-20% for other uses such as fibres, biofuel, timber) [46,59]. Green water consumptive use estimates for agriculture vary widely, mainly due to varying accounting methods of evaporation (i.e. including throughout the year or only during the growing season); and whether grazing lands are included [46,60],

and subsequently whether all grassland biomass is included or only the actively grazed portion [46,61]. Here we have excluded the higher end of the range estimates from [61] (8,155 $km^3 yr^{-1}$) and [46] (9,560 $km^3 yr^{-1}$) that include evaporation from all grazing lands, to calculate the total blue and green water consumptive use for agriculture. Here, we used the novel control variable of the freshwater change PB for green water, and estimated the food system contribution to green water change by evaluating rootzone soil moisture availability changes on agricultural land only. We use pre-industrial (1691-1869) and historical (1840-2005) monthly rootzone soil moisture availability simulations at 0.5 degree spatial resolution from LPJmL v4, a global dynamic vegetation model, representing biogeochemical land surface processes including water fluxes [62]. Rootzone soil moisture data ('rootmoist') derive from ISIMIP 2b simulations, using four different GCMs (HadGEM2-ES, GFDL-ESM2M, IPSL-CM5A-LR, MIROC5), available at https://data. isimip.org (accessed 05 February 2025). These simulations only run to 2005 and are therefore do not present to most recent available data, but is consistent with the freshwater change PB assessment from [52]. We use HYDE3.2 [29] to identify 'agricultural land' based on cells >10% coverage of crop and/or pasture land in the year 2010, internally consistent with the land use input data for the LPJmL simulations. All other land area (including ice-lands) are ignored. We calculate the agricultural land and total land area (%) departing from local pre-industrial variability envelopes monthly from 1900 to 2005 (**Supplementary Figure 3a**), and calculate dry and wet departures distinctly (**Supplementary Figure 3b**) using the *boundaries* R package [63]. Ensemble mean transgression rates and ensemble range (between brackets) are based on a ten-year average (1996-2005). We find that most recent (i.e. 2005), 16.75% (ensemble range: 15.5 – 18.9) of the agricultural land is transgressed. This corresponds well with the global average transgression rate (all land excluding permanent ice) of 17.2% (ensemble range: 16.2-19.5)[1], suggesting that agriculture does not appear a persistent, multiplying stressor on green water compared to other drivers, such as climate change, at least when globally aggregated. However, these transgressions may mask specific regional occurrence of wet departures (from irrigation) or dry departures (-from agricultural-induced land degradation or soil erosion) associated with agriculture, as well as regional strong deviations that may occur from agricultural land use. Further growth of green water transgressions in the absence of climate mitigation in the future is expected [65]

[1] Porkka et al. (2024) estimates the total global land area beyond local variability envelopes being 16.6% from LPJmL (using the same set of GCMs). The difference (0.5%) can be explained by slightly deviating method in calculating the PB status between Gerten et al. (2024) and Porkka et al. (2024). This deviates from the ensemble mean (15.8%) provided in [52] that is used in **Table 1.**

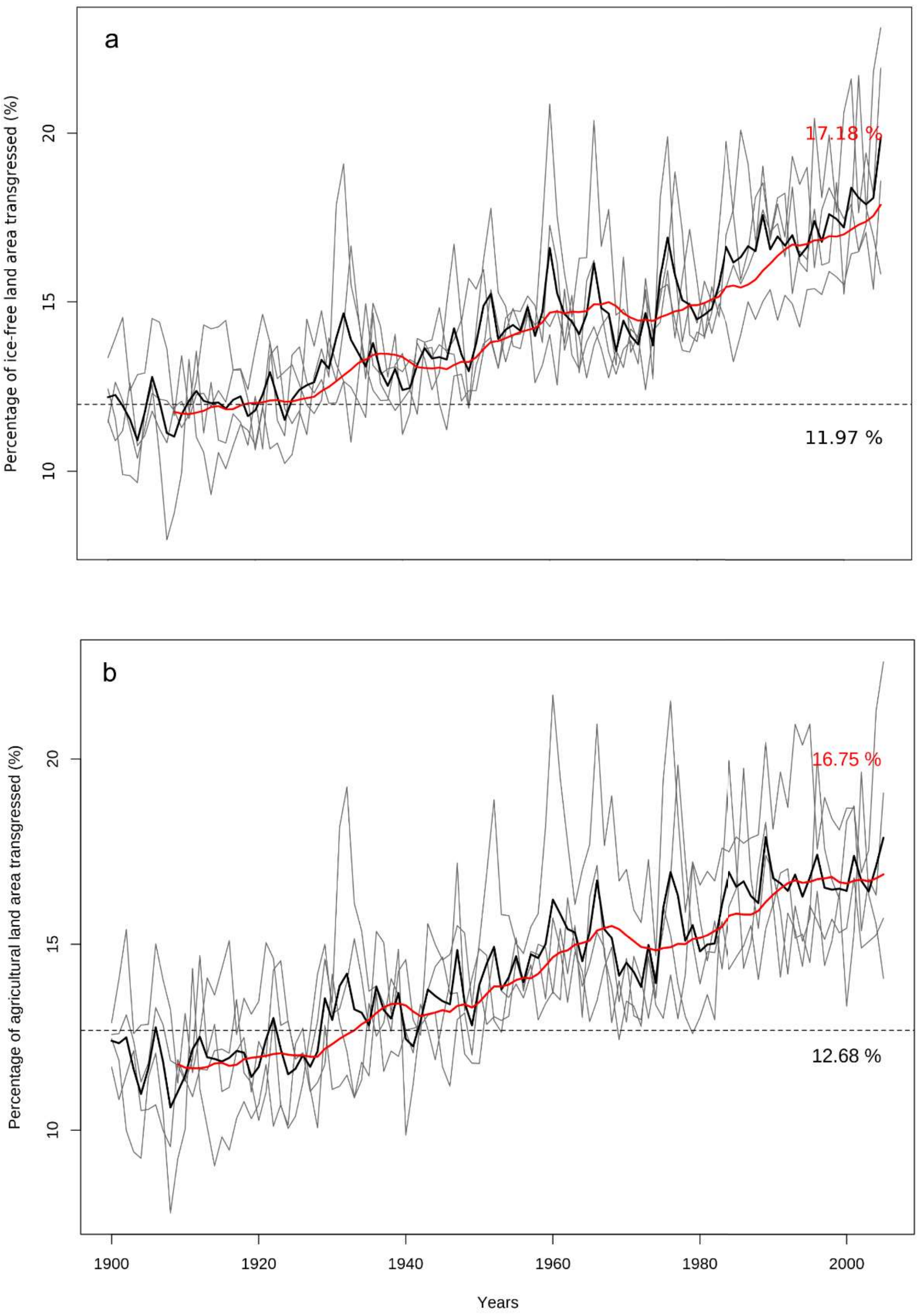

Supplementary Figure 3 Timeseries of the global land area with local deviations from pre-industrial 5th—9th percentile variability envelopes. Simulations derive from ISIMIP 2b-LPJmL data. Grey lines represent the four GCMs that we used for forcing (HadGEM2-ES, GFDL-ESM2M, IPSL-CM5A-LR, MIROC5). The black line represents ensemble mean; the red line the 10-year moving average. The dotted line represents the upper end (95th percentile) of the global pre-industrial variability envelope. **a**) All ice-free land, of which 17.18% is transgressed. **b)** agricultural land only (excludes all areas with <10% agricultural area), of which 16.75% is transgressed.

Supplementary Table 6 Overview of literature reporting on present-day blue and green water use for agriculture (upper rows) and total water use (lower rows) to estimate the contribution of agriculture to blue water withdrawals, and blue and green water consumption ($km^3\,yr^{-1}$).

AGRICULTURE					
**Reference**	**Blue water**		**Green water**	**Blue & green**	**Approach/data**
	Withdrawal ($km^3\,yr^{-1}$)	***Consumptive use ($km^3\,yr^{-1}$)***	***Consumptive use ($km^3\,yr^{-1}$)***	***Consumptive use ($km^3\,yr^{-1}$)***	
[46]	n.a.	1,269 from cropland	7,670 from cropland; 1,690 from pastures (grazed biomass)	10,630 (4,564 from cropland during growing period)	FAO Balance Sheets and LPJmL v4. Comprises ET during growing and fallow period. Excludes biomass on pastures that is not grazed (additional 9,560 $km^3\,yr^{-1}$)
[54]	2,700 (1,500—3,700)	1,200 (1,040—1,370, incl. 545 from groundwater	n.a.	n.a.	Irrigation use from hydrological model ensemble (represented by the range)
[36]	n.a.	1,810	n.a.	n.a.	Country-specific crop blue water footprints (2010) from IMPACT model.
[55]	70%	84%	n.a.	n.a.	Based on [56]
[59]	n.a.	n.a.	5,700 from cropland 2,900 from livestock grazing	n.a.	Water Footprint estimates [66]
[67]	n.a.	1,180 (irrigation)	Rainfed crops: 4,586 Irrigated crops: 919	6,685 (crop water use)	GCWM computing daily ET for 26 crops (1998-2002). Also included in ensemble presented in [54]
[68]	n.a.	1,530 km^3 (irrigation)	Rainfed crops: 7,820 Irrigated crops: 1,720	11,070	Global hydrology simulation (H08) (1985-1999).
[60]	n.a.	1,228 (927-1,530) (irrigation)	6,371-9,823 (rainfed and irrigated, full year)	n.a.	Based on intercomparison of 7 global hydrology models, represented by the range.
[61]	2,555	1,364	7,200 (cropland) 8,155 (grazing land)	8,564 - 16,719	Global dynamic biosphere model (LPJmL). Higher end of the range includes grazing land.
[51]	2,409	n.a.	n.a.	n.a.	Global dynamic biosphere model (LPJmL). Also included in ensemble presented in [54]

**Estimated range (rounded)**	**2,400—2,700**	**1,180—1,800**	**Cropland: 5,500—9,600 Grazing land: 1,700—2,900**	**8,400—14,300**	

**TOTAL**					
**Reference**	**Blue water** *Withdrawal (km³ yr⁻¹)*	*Consumptive use (km³ yr⁻¹)*	**Green water** *Consumptive use (km³ yr⁻¹)*	**n.a.** **n.a.**	**Approach/data**
[59]	n.a.	n.a.	Total: 72,000		Total green water flow (including natural flows)
[69]	3,800	n.a.	n.a.		n.a.
[50]	n.a.	1,700—2,270	n.a.		From existing literature. Range based on [56]; [68]. PB definition based on 5 approaches to calculate EFR
[56]	n.a.	2,600	n.a.		n.a.
[70]	2,376	1,831	n.a.		Based on modeled agricultural, industrial and domestic water demand.
[68]	n.a.	n.a.	Total: 72,080		Global hydrology simulation (1985-1999)
[51]	3,480	n.a.	n.a.		Global dynamic biosphere model (LPJmL) simulations. Includes agriculture, household, industry and livestock.
[71]	n.a.	1,700	n.a.		GCWM and FAO database. 75% is agricultural related. 21% is for reservoir management, of which 69% is for energy use. Food contribution is thus at most 81.5%
**Estimated range**	**3,480—4,000**	**1,831—2,600**			
**Food system (%)**	**% 60—77%**	**% 45—98**	**% 11-20**		

Food system boundary for freshwater

Willett et al. (2019) proposed a boundary for freshwater consumption of food systems of 2,500 km^3 yr^{-1}. They adopt the global value of 2,800 km^3 yr^{-1} by [50], with an assumed ~90% allocation to food production for 2050, based on the (high end of the) agriculture share estimate (75-84%) of global consumptive water use [55] and assuming that industrial and domestic water consumption can also be limited in the future. Given an estimated agricultural water consumption of ~1,400–1,800 km^3 yr^{-1}, they suggest that the FSB is not yet overstepped [16]. A related study estimates that the present-day total consumptive water use (±2,600 km^3 yr^{-1}) compared to the consumptive water use from agriculture (estimated 1,810 km^3 yr^{-1} – at the higher end of the range) [36], suggests that agriculture is currently responsible for 70% of the total consumptive water use. Accordingly, scaling the PB range provided by Gerten et al. (2013) provides a stricter boundary of 1,980 (780-3,190) km^3 yr^{-1} [36], which implies that the food system is moving close to boundary transgression.

For blue water, we reinstate earlier boundaries based on river-basin EFR [36,50,51] but propose a stricter boundary than [16]. There are two reasons that support a more precautious approach. First, we consider the lower end of the agricultural consumptive use estimates provided in (**Supplementary Table 6**) which suggests that agriculture is currently responsible for less than 84% of the total consumptive water use. Using the 70% consumptive use estimated provided in [36] suggests a boundary of 1,980 km^3 yr^{-1}. Second, the uncertainty range of the freshwater PB (1,100-4,500 km^3 yr^{-1}) suggests that the lower end of the range the boundary is already transgressed. Together, these aspects support the proposal of a stricter boundary, where food systems remain below 2,000 km^3 yr^{-1} of consumptive water use. This includes the use of groundwater, of which pumping should regionally remain below recharge levels [33]. Regional assessments show that these EFR budgets along various rivers systems are already transgressed due to a range of pressures [35,50]. [51] find that around 40% of the current global irrigation water withdrawals (~2,400 km^3 yr^{-1}) are transgressing local EFRs, summing up to a global EFR deficit of 997 km^3 yr^{-1}. Reducing irrigation water use to respect EFRs in these regions would lead to yield losses of more than 10% in more than half of the worlds' irrigated croplands.

For green water, critical knowledge gaps remain regarding the setting of a FSB. In addition, the current PB definition is based on pre-industrial variability envelopes but limited evidence supports this particular variability range in light of emerging risks to Earth system stability. We therefore preliminarily suggest that agricultural lands (currently 16.8% of agricultural land is transgressed), should return back to the global variability envelope (11.1%).

Supplementary Table 7. Overview of literature on the Freshwater planetary boundary and food system boundaries, and current status.

Reference	Planetary boundary	Food system boundary	Current status
[21]	4,000-6,000 km^3 yr^{-1} of consumptive blue water use	n.a.	Withdrawals: 4,000 km^3 yr^{-1} (based on [69]. Consumptive use: 2,600 km^3 yr^{-1} (based on [56]
[50]	2,800 (1,100-4,500) km^3 yr^{-1} of consumptive blue water use	n.a.	>1700-2270 km^3 yr^{-1}(based on [56] and [68]
[37]	Global: 4,000 km^3 yr^{-1} of consumptive blue water use<br><br>Water basin: 25-55% depending on low/high flow months water withdrawal	n.a.	2,600 km3
[55]	2,800 km^3 yr^{-1}	*84% (based on current use)*	Adopting control variable from [37] and boundary from [50]
[36]	2,800 km^3 yr^{-1} (range 1,100–4,500 km^3 yr^{-1}) of consumptive blue water use	1,980 km^3 yr^{-1} of consumptive blue water use	Basin-level boundaries based on (stringent) EFRs (range), scaled to (current) agricultural blue water use (estimated at 70%)
[16]	2,800 km^3 yr^{-1} of consumptive blue water use	2,500 km^3 yr^{-1} of consumptive blue water use	Total consumptive use: 1800-2100<br>Food system contribution: 1400-1800<br>Based on [70]; [56].<br>Current use of 75-84%; allocated 90% towards 2050
[35]	Restricting human freshwater use (withdrawal: 25–55%, 40–70% and 55–85% of mean flow in low-, intermediate- and high-flow months, respectively)	Same as planetary boundary (2,800 km^3 yr^{-1})	n.a.
[52]	Blue water: global land area (%) with human induced disturbance of blue water flow: 10.2%<br><br>Green water: global land area (%) with human-induced disturbance of water available to plants (% land area with deviations from pre-industrial variability): 11.1%	n.a.	Blue water: 18.2%<br>Green water: 15.8%

Supplementary Text 5. Biogeochemical Flows

Supplementary Text 5a. Nitrogen: Planetary boundary and control variable

We use the N surplus as control variable, deviating from the external N input control variable in [3]. Critiques, already signalled by [72] and further elaborated by [73] for nitrogen are that: (i) global limits for intentional N fixation are affected by changes in Nitrogen Use Efficiency (NUE), (ii) and do not account for losses from recycled N sources and (iii) only included notions of possible reduction of N losses without considering regions where increases are plausible without negative environmental impacts (but with positive yield impacts). More details are given in [74].

We use the recently derived N surplus boundary by [73] since their methodology explicitly accounts for critical environmental impacts from N losses to the environment, and includes both agricultural and non-agricultural sources (i.e. sewage and N runoff from natural land) which can sometimes exceed agricultural N sources (see extended data from [73].

Based on simultaneous protection of terrestrial and aquatic biodiversity, and groundwater quality, [73] derive a planetary N surplus boundary of 57 Mt N yr^{-1}, being half of the estimated current (2010) N surplus of 119 Mt N yr^{-1}. More specifically, they derive local N surplus boundaries based on three impacts: (i) eutrophication and acidification of terrestrial ecosystems by NH_3 emissions based on critical N deposition levels (varying from 7.5—10 kg N ha^{-1} yr^{-1}, depending on the ecosystem) (ii) eutrophication of aquatic ecosystems by N runoff, based on critical N concentrations in surface water (2.5 mg N L^{-1}) and (iii) groundwater contamination by NO_3 leaching, based on a critical NO_3 concentration (50 mg NO_3 L^{-1}) used by the WHO drinking water standards. Local boundaries are subsequently based on the N surplus value associated with the most constraining critical value for surface water load, groundwater leaching or deposition, assuming current NUE levels (see [73] for details). Surface water quality was generally the most constraining factor affecting the N surplus boundary. Increasing NUE increases the critical N input without affecting the critical N surplus. Assuming proportional changes in non-agricultural N losses only marginally increases the N surplus boundary for agriculture with current NUE levels [73]; we therefore propose to remain with the proposed 57 Tg N yr^{-1} surplus boundary for food systems.

Agricultural-induced N inputs comprise N fertilizer (91 Tg yr^{-1}), biological N fixation (30 Tg N yr^{-1}), manure (78 Tg N yr^{-1}) and NH_3 and NOx deposition (23 Tg N yr^{-1}) from manure and fertilizer emissions (37 Tg N yr^{-1}), including emissions from extensive grasslands [73]. The total agricultural N input amounts 233 Tg N yr^{-1}.

Supplementary Text 5b. Phosphorus: Planetary boundary and control variable

For P, we suggest using P delivery to surface water as the control variable, comprising of P losses from agriculture, aquaculture, and waste water and natural losses. P loss from the soil is the sum of runoff and erosion, being the dominant source since particulate P input to surface water is the largest source of P. Erosion is determined by (i) the soil erosion rate and (ii) the soil P content (soil P pool), which is affected by the natural soil P content and the legacy of accumulated P over time [76] being a reason for not using P fertilizer as a control variable. The acceptable P delivery to water in a region can be derived by multiplying a *critical P concentration* with water fluxes, while accounting for P river retention [74]. The global discharge is estimated at 38,000 $km^3 yr^{-1}$, and using a critical P concentration of 0.075 mg P L^{-1}, [77,78] and a global average river P retention fraction of 53% [79], this leads to a critical global P delivery of ca**.** 6.1 Tg P yr^{-1}, compared to the current river P delivery to surface water of 9.7 Tg P yr^{-1} [74].

Supplementary Text 5c. Present-day contribution of food systems

We define the contribution of the food system to present-day N and P losses to surface water based on the contribution of agriculture, aquaculture and wastewater N sources to the current N and P delivery. We estimate food systems contributions of 72% for N (51 Tg N yr^{-1}) and 74% for P (7.1 Tg P yr^{-1}) (**Supplementary Table 8**). Unlike P, N also contributes to other environmental issues, including terrestrial biodiversity loss through N deposition (52%, or 11.9 Tg N yr^{-1}) and (drinking-)water contamination through leaching of nitrate to groundwater (81%, or 45 Tg N yr^{-1}), as explained in **Supplementary Table 8.**

Note that the contribution of N_2O was not considered in the setting of the N surplus boundary [73]. Nitrous oxide is a strong GHG, and fertilizer and manure application are responsible for 75% of the N_2O emissions. However, N_2O emissions currently contribute only 7% of the total forcing of all GHG (IPCC, 2022), and the abatement potential of N_2O is lower compared to other gases. Remaining with the proposed N boundary of 57 Tg of annual N surplus, will reduce N_2O emissions from the required regional reduction of N inputs from agriculture, by 55% [73].

Supplementary Table 8. The contribution of the food system to present day N and P losses.

Specific sources	Current NH_3 emission to air Tg N yr^{-1}	Current N delivery into surface water Tg N yr^{-1}	Current P delivery to surface water (Tg P yr^{-1})	Current NO_3-N leaching Tg N yr^{-1}
**Agriculture**	37[1]	37[4]	5.3[6]	45[8]
**Aquaculture**	-	1.8[4]	0.19[6]	-

**Wastewater**	-	12[4]	1.6 (*17% of total delivery from sewage)*[6]	-
**Non-food (nature and other sources)**	-	20[4]	2.45[6]	-
**Total**	Deposition on natural land (NH_3+NO_X): 23[2]	71[4]	**9.7[6]**	56[8]
Share food systems	***52%[3]***	***72%[5]***	***74%[7]***	***81%[9]***

[1] The emissions from agriculture (37 Tg N yr^{-1}) include grasslands. Excluding grasslands yields 30.8 Tg N yr^{-1}.

[2] Data from [80] for forests, being the dominant non-agricultural terrestrial ecosystem. Deposition from NH_3 estimated 11.9 Tg yr^{-1}; Deposition from NO_X estimated 11.1 Tg yr^{-1}.

[3] The share of agricultural N emissions to biodiversity impacts is estimated as the ratio 'NH_3 deposition/NH_3+NO_X deposition' on terrestrial ecosystems, being 11.9 Tg N yr^{-1} over the total 23 Tg N yr^{-1}, amounting to 52%. The contribution assumes that all NH_3 comes from agriculture (although a small part comes from non-agricultural sources) and all NO_X from non-agriculture (although a small part comes from agricultural sources). Note that using the agricultural share of NH_3+NO_X emissions is less appropriate since a large part of NO_X is deposited on marine systems.

[4] Data from [79] on the N delivery at global scale for the year 2015.

[5] The contribution of food systems to surface water load is defined as the share of agriculture, aquaculture and waste water N delivery (51 Tg N yr^{-1}) over the total N delivery (71 Tg N yr^{-1}), amounting to 72% from agricultural sources.

[6] Data based on [79] and from [81].

[7] The contribution of food systems to surface water load is defined as the share of agriculture, aquaculture and human waste P delivery (7.1 Tg P yr^{-1}) over the total P delivery (9.7 Tg P yr^{-1}), amounting to 74% from food systems.

[8] Data from [73] on the N leaching at global scale for the year 2010.

[9] The contribution of agricultural sources to N leaching is calculated as the ratio N leaching from agricultural land (45 Tg N yr^{-1}) dived over N leaching from all land (56 Tg N yr^{-1}), leading to 81% due to agricultural N sources.

Supplementary Text 6. Stratospheric ozone

The planetary boundary for Stratospheric Ozone Depletion protects all life from harmful UV radiation and its boundary is set as 276 Dobson Units (DU), allowing for a <5% deviation from preindustrial levels. Various substances can destroy ozone in the Earths' stratosphere, their strength measured through their respective Ozone Depleting Potential (ODP), i.e. the amount of ozone destroyed per unit mass of substance released at the Earth surface, relative to CFC-11. Nitrogen oxides (NO_X) catalytically break down stratosphere ozone, and surface N_2O emissions are currently the main source of stratospheric NO_X [82], contributing 180 kT yr^{-1} ODP weighted emissions in 2008.

N_2O emissions have increased with 40% since 1980, mostly from direct agricultural emissions from nitrogen additions, with anthropogenic emissions now amounting 7.3 Tg of N yr^{-1} (**Supplementary Table 9**). Direct agricultural N_2O emissions derive from application of nitrogen fertilizer and manure (soil emissions), manure on pasture and rangelands, manure management and aquaculture [4]. Direct soil emissions from fertilizer and manure are the largest component (2.1 Tg N yr^{-1}), followed by manure on pasture (1.4 Tg N yr^{-1}) and aquaculture (0.12 Tg N yr^{-1}) and manure management (0.26 Tg N yr^{-1}). These estimates suggest that direct agricultural emissions comprise around 3.9 Tg N, or 54% of the total anthropogenic N_2O emissions [4]. This is lower than AFOLU emission in [1] – 6.6 N_2O, i.e. 4.2 Tg N yr^{-1} , or 69% of total net anthropogenic emissions – because [4] excludes biomass burning.

Supplementary Table 9: Annual (anthropogenic) N_2O emissions in 2020. Direct anthropogenic emissions include sources from fossil fuel burning, industry, waste(water) and biomass burning; indirect anthropogenic sources include emissions from nitrogen additions coming from land and ocean N deposits, and anthropogenic-derived surface water loads. Note that the first estimates (mean and range) of the total emissions (natural and anthropogenic sources) in the right-most column, are based on a bottom-up inventory-based approach, while the second range of estimates are based on a top-down atmospheric measurement inversion approach. Note that these emissions represent N_2O-N (Tg N yr^{-1}) fluxes; conversion to N_2O (Tg N_2O yr^{-1}) requires conversion with factor 1.57. Data from [4]

Direct agricultural emissions (Tg N yr^{-1})	Direct anthropogenic sources (fossil fuel, industry, waste(water), biomass burning (Tg N yr^{-1})	Indirect anthropogenic sources (Tg N yr^{-1})	Total anthropogenic N_2O emissions (Tg N yr^{-1})	Total emissions (natural and anthropogenic) (Tg N yr^{-1})
3.9	2.1	1.3	7.3	Bottom-up: 18.5 (10.6-27.0)<br>Top-down: 17.0 (16.6–17.4)

Supplementary Text 7. Ocean Acidification

Planetary boundary and control variable

The planetary boundary for Ocean Acidification is expressed in terms of its aragonite saturation state (Ω_{arag}). Over the past decades, a quarter of the increase in human-induced atmospheric CO_2 emissions has been taken up by the ocean [83], which increased the concentration of free H+ ions in ocean surface water (lowering the pH), and subsequently changes the carbonate chemistry. It has lowered Ω_{arag}, which is crucial for the formation of shells by many marine organisms. Aragonite dissolves at a saturation state < 1. The PB suggests that therefore ≥80% of the aragonite saturation state pre-industrial average (3.44 Ω_{arag}) should be preserved [21,37], which corresponds to 2.75 Ω_{arag}. The current state is 2.8 Ω_{arag}, so while we are within the PB for ocean acidification, we are moving rapidly towards the zone of increasing risk. The rate of change in pH is a hundred-fold faster than known background rates from the past 20 million years [21] and these changes are expected to continue over the future with rising CO_2 concentrations. Even in the most optimistic future scenarios (SSP1-1.9), atmospheric CO_2 concentrations are expected to increase up to 430-450 ppm to 2050 [84]. Regional effects of acidification are already affecting marine life, as well as polar oceans and deep oceans that are more sensitive to acidification (i.e. low water temperature implies higher uptake of CO_2, and hydrostatic pressure within large depth greatly decreases Ω_{arag}, so that a change in Ω_{arag} will be more detrimental for marine organisms in deeper layers of the ocean compared to the surface [85]. Although global changes in ocean pH can be predicted roughly by atmospheric CO_2 concentrations, regionally, additional drivers along coastal zones (i.e. nutrient run-off, mostly from food) will likely lead to regionally higher levels of acidification [86] and thus a higher share of the food systems contribution to regional acidification rates.

Present-day contribution of the food system to ocean acidification

CO_2 emissions from food systems comprise around 60% of the total food-related GHG emissions (in terms of CO_2-equivalent, see [2]; the rest being non-CO_2 emissions such as CH_4 and N_2O. Most CO_2 emissions derive from land conversion (5.9 Gt CO_2 yr^{-1}, **Supplementary Table 2**); the remaining are pre- and post-production emissions deriving from supply chains (4.3 $GtCO_2$ yr^{-1}), food transport (0.5 $GtCO_2$ yr^{-1}), and waste disposal (0.03 $GtCO_2$ yr^{-1}) [2]. Total global CO_2 emissions in 2019 (consistent with time period of food system estimates mentioned above) amounted 45 (±5.5) Gt CO_2 yr^{-1} [84] which implies food systems are responsible for a quarter of the driving force behind worsening patterns of ocean acidification. Historical emissions from land use change (dominated by agricultural expansion) between 1750 and2022 also suggest that cumulative emissions have contributed to around 25% of

the total cumulative carbon emissions [24] of which over time, a major part has been taken up by the ocean. Both cumulative land use emissions and the recent decadal CO_2 emissions from food systems (including pre- and post-production emissions) converge around 25% contribution of food systems to CO_2 emissions and associated ocean carbon uptake.

Food system boundary

Restating the climate boundary for food systems, we argue that the CO_2 emissions should be reduced to zero to keep global warming below 1.5 (50%) or 2 (66%) degrees of warming, and halt further CO_2 uptake and subsequent change in the aragonite saturation state in oceans. [1] show that mitigation potential of non-CO_2 gasses is relatively low, suggests that most CO_2 emissions should be emitted to remain within the Climate change boundary for food systems (5 $GtCO_2e$ yr^{-1}). Remaining within the boundary for land use (i.e. no new land conversion) eliminates the CO_2 emissions from land conversions. Furthermore, assuming full decarbonization of the energy sector eliminates remaining emissions from post-production that derive for almost 90% from supply chain energy use [2].

Supplementary Text 8. Aerosol loading

Planetary boundary and control variable

The planetary boundary for aerosol loading is based on aerosol optical depth (AOD), which broadly captures the reduction in sunlight reaching the Earth's surface due to the increase in air pollutants from both natural sources (i.e. desert dust, wildfires) and anthropogenic sources, such as agriculture, transport and energy generation. Aerosol loading from anthropogenic sources has increased, while dust deposition has also doubled since 1750. Due to the varying impact of AOD on regional ecosystems and climate, setting a global boundary that is regionally applicable is challenging. [3] therefore propose to express the control variable as the annual mean interhemispheric difference in AOD, which can affect various monsoon systems by shifting the location of the Intertropical Convergence Zone. The current status of the PB is based on satellite imagery measuring aerosol optical depth from 94 different sources, including satellite data, climate-model ensembles and re-analyses [87]. The PB is set at a difference of 0.1, with a high uncertainty of the zone of increasing risk at 0.25.

Present-day contribution of food systems

Identifying the contribution of food systems to the interhemispheric difference in AOD is challenging for various reasons. Changes in AOD are dependent on the concentration of a wide range of atmospheric particles with different optical characteristics, i.e. nitrogen components (NH_3, NOx and N_2O), SO_2, CO, organic compounds and particulate matter (PM). Globally, food systems are responsible for ~10-90% of various emissions (**Supplementary Figure 4**). The atmospheric concentration of aerosols (measured in fine particular matter with a diameter of less than 2.5 µg, $PM_{2.5}$) is the result of pollutants from natural and anthropogenic sources. Natural sources (including inorganic ions, BC, OC and dust) comprise around 75% of the total particulate matter mass in the atmosphere (based on 2030 projections from [27], although anthropogenic activities can increase the emissions of sources considered natural, such as dust and sand from land degradation [88]. $PM_{2.5}$ concentrations derive from primary $PM_{2.5}$ sources and secondary emissions, which are formed through complex atmospheric interactions of emitted precursors such as NH_3, NO_X, SO_2 and organic compounds (VOC) [25]. Primary $PM_{2.5}$ emissions attributed to food systems (including production, energy, processing and waste emissions) are estimated to comprise almost a third (28%) of primary anthropogenic $PM_{2.5}$ emissions [23]. Higher estimates (up to 58% of primary anthropogenic $PM_{2.5}$ attributed to food from [25] may be explained by uncertainties in the $PM_{2.5}$ emissions from land conversion [23]. The majority of primary $PM_{2.5}$ from food systems derive from biomass burning (land conversion and agricultural waste burning) [25]. Secondary $PM_{2.5}$ is formed from NH_3 (72-86% of global

NH_3 derives from agriculture)[23,25,73] and NO_X emissions (combustion), which form nitrate aerosols, mainly ammonium sulfate, ammonium bisulfate and ammonium nitrate [89]. Secondary $PM_{2.5}$ forms a significant part of $PM_{2.5}$ concentrations in some regions (i.e. 50% in Europe, [90], but specific (sectoral) attribution of secondary $PM_{2.5}$ concentrations is challenging. The relationships between agricultural emissions and $PM_{2.5}$ levels will likely change in a warmer climate, given the temperature-volatility relationships for several of the nitrogen compounds (Wang, Chen, Isaksen, Noone, & McGuffie, 2011).

Various studies show that the spatial and temporal distribution of aerosol loading is highly variable, and characterized by a strong regional difference in drivers, including between hemispheres. Food system emissions contribute 22% (Southern Asia), 50% (US) to 53% (Europe) to total $PM_{2.5}$ concentrations [23]. In Northern Africa, natural sources of $PM_{2.5}$ predominate, mostly comprising dust from the arid zones of the Sahara and Middle-East. Across the northern hemisphere, agriculture appears to be the predominant source of $PM_{2.5}$ concentrations, particularly from ammonia [73,89]. In the southern hemisphere, a large share of the $PM_{2.5}$ concentrations is traced back to biomass burning (including both wild-fires and prescribed burning for land conversion, agriculture waste burning), releasing mostly primary $PM_{2.5}$ [25,91]. Although the global contribution of biomass burning to $PM_{2.5}$ concentrations is relatively small (5%), it can regionally constitute 70-90% of $PM_{2.5}$ concentration [91]. Data analysis based on Global Fire Emission Database suggest land conversion is the most important source of all food-attributed $PM_{2.5}$ concentrations, emitting in total 14 million tonnes yr^{-1} (in 2012), 65% of all primary $PM_{2.5}$ emissions from food [25]. The Global Wildfire Information System reports that in the same year, all wildfires emitted 32 million tonnes of $PM_{2.5}$, suggesting that around half of the fire-related $PM_{2.5}$ emissions can be attributed to agricultural land conversion. In addition, agriculture waste-burning emits around 7 Mt $PM_{2.5}$ yr^{-1}, suggesting that the share of food systems (here considered as land conversion and agricultural waste burning) to global $PM_{2.5}$ emissions from biomass burning is at least 50%. Primary $PM_{2.5}$ emissions from the production phase of agriculture is negligible [25]. In the southern hemisphere, where most of the land conversion is concentrated (i.e. tropical forests of the Amazon, Congo, Indonesia, [39], the food system share can even be higher than 50%.

It is conceptually challenging to express the share of food systems in terms of the *interhemispheric difference* in AOD, which can only be determined based on total aerosol loading in both hemispheres (including all sector-based contributions). Due to a wide range of pollutants from different sources that interact in the atmosphere, and the interhemispheric difference in drivers, there is no single quantification of the proportion of aerosol loading and subsequent AOD change can be attributed to food systems. Therefore, we quantify the contribution of food systems to $PM_{2.5}$ concentrations in

the northern hemisphere based on the *emissions* of NH_3 as main contributor to $PM_{2.5}$ concentrations [91]. For the southern hemisphere we quantify the contribution of food systems to $PM_{2.5}$ concentrations from biomass burning as the main source of $PM_{2.5}$ concentrations [91].

Staying within the N surplus boundaries (**Table 1**) reduces NH_3 emissions from agriculture with 45% compared to current levels (from 37 to 20 Tg N yr^{-1}) [73], particularly in the northern hemisphere where these local N transgressions are concentrated (**Figure 4**). Dietary changes and a reduction of FLW can reduce food-based ammonia emissions with 44—84% [27] (range represent lower end of estimates for dietary shifts with different ambition levels, i.e. flexitarian vs. vegan) and 10.2% [92], respectively, and subsequent anthropogenic $PM_{2.5}$ concentrations in the northern hemisphere with 52 tonnes yr^{-1} (7%) (**Supplementary Figure 5**). We therefore suggest ammonia emission reductions of at least 45% (<20 Tg N yr^{-1}) to remain within the N surplus boundary. Second, biomass burning from land conversion, particularly prominent in the southern hemisphere, should be halted in line with the Land System Change boundary, effectively reducing $PM_{2.5}$ from biomass burning by half. However, it is uncertain how much reduced NH_3 emissions and biomass burning subsequently reduce $PM_{2.5}$ concentrations across hemispheres, and the interhemispheric difference in AOD, due to uncertainties in the exact contribution to NH3 emissions to the AOD; as well as the contribution of biomass burning to AOD. In addition, evidence suggest that biomass burning is increasingly driven by climate change [93].

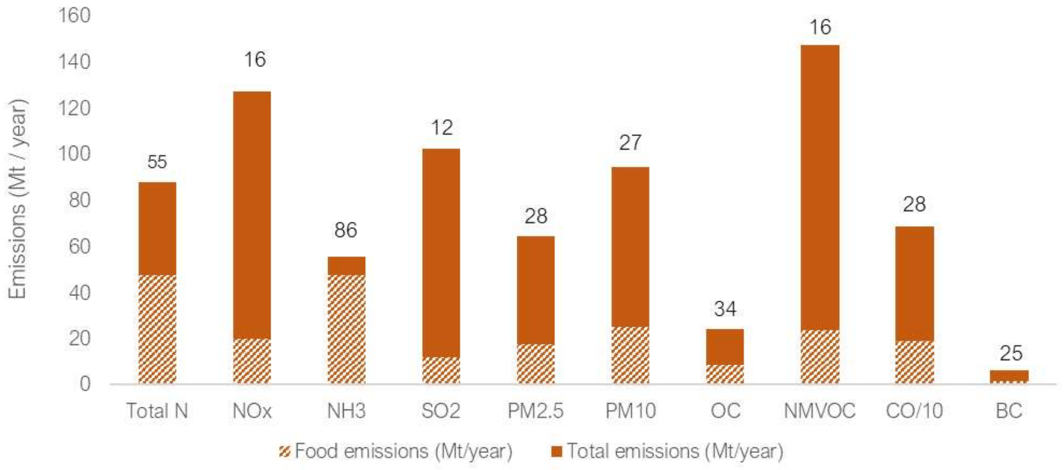

Supplementary Figure 4. Food system emissions and total emissions of 9 substances and total N (comprising the N equivalent of NOx, NH_3 and N_2O). Data derives from the Emissions Database of Global Atmospheric Research (EDGAR-FOOD-AP) v6.1. Numbers on top of bar chart present the relative shares of the food system emissions (%). Note that CO emissions are divided by 10 for display reasons. EDGAR-FOOD database is based on estimated country-specific emissions that includes production, processing, distribution, consumption and

waste over 1970-2018. An elaborate description of the processes included for each of the pollutants is provided in [23] Note that $PM_{2.5}$ and PM_{10} includes only primary pollutants.

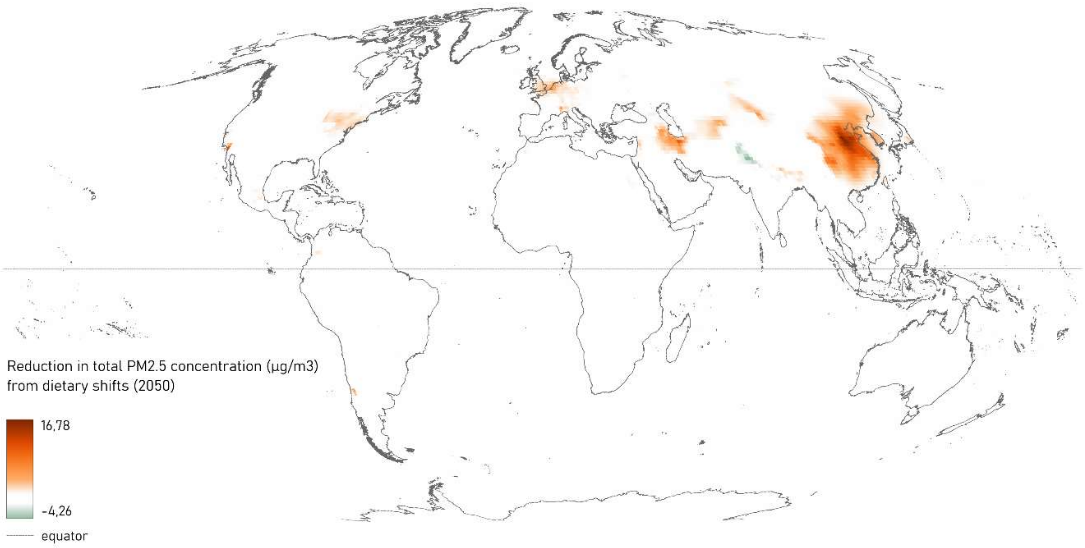

Supplementary Figure 5 Reduction in total $PM_{2.5}$ concentration (µg m^{-3}) from a shift to vegan diets (compared to business as usual projections) for the year 2050. Dotted line represents the equator separating northern and southern hemisphere. Emission data derives from [27] and is based on CAPRI agriculture-economic model projections based on a middle-of-the-road development pathway (SSP2) and the associated $PM_{2.5}$ emissions trajectory (RCP 6.0); emission trajectories are subsequently estimated using TM5-FASST air quality model. All changes smaller than 1% (both above and below 0) are marked out from this map (i.e. indicated in white in the legend color bar) to reduce noise. The (local) increase in emissions from a shift to vegan diets (in green in South Asia) is likely due to an increase in fertilizer use.

Supplementary Text 9. Novel entities

Planetary Boundary definition and control variable

The novel entities boundary in the planetary boundaries framework refers to entities that are novel in a geological sense, which could have large-scale impacts that threaten the integrity of Earth system processes. These include the release of synthetic chemicals and substances, such as microplastics, endocrine disruptors, and organic pollutants; anthropogenically mobilized radioactive materials; and genetically modified organisms and other evolutionary modifying processes [3]. These entities are intentionally and unintentionally manufactured chemicals, engineered materials, as well as their transformation products. The planetary boundary for Novel Entities sets the limit as the share of released chemicals with adequate safety assessment and monitoring as a candidate control variable. [94] suggest that 'for novel entities introduced exclusively by humans (e.g., xenobiotic organic chemicals, plastics), by definition there is no "natural variability" (i.e. pre-industrial baseline) against which a control variable can track change on human time scales, nor is there a biophysical precedent for identifying thresholds'. Furthermore, novel entities can have a wide range of effects through various environment pathways, affecting other PB processes. Their potential high risks to biosphere integrity support a strict precautionary approach, and therefore, [3] set the boundary at a 0% release of chemicals without adequate safety testing. Here, we consider two novel entities sub-variables that are critical in food systems: pesticide application for crop protection, and antimicrobial use in the livestock sector. Both entities are associated with considerable threats to ecosystems and human health [95–97]; yet their use has dramatically increased over the past decades [98], posing critical risk to Earth system stability.

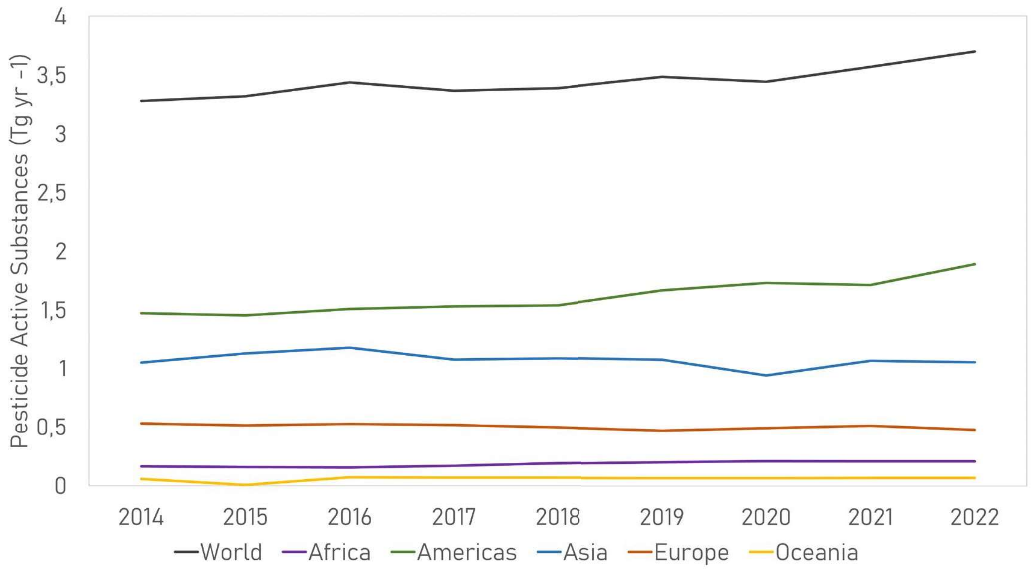

Supplementary Figure 6: Total pesticide application between 2014-2022. Data from FAOSTAT (2024).

Supplementary Text 9a. Pesticide application as an indicator for the food system contribution

Agriculture accounts for at least 85-90% of the total pesticide usage, including herbicides, fungicides and insecticides [99]. Global pesticide application amounted to 3.3—3.7 Tg of PAS yr^{-1} between 2014—2022 (**Supplementary Figure 6**). Synthetic chemical pesticides are exclusively introduced by humans, and pose a risk to the health of non-target organisms [100], including humans [101]. Therefore, we adopt *pesticide application* as an indicator for the food system contribution to the Novel Entities planetary boundary. More specifically, we use local pesticide pollution risk emerging from Pesticide Active Substances (PAS) residues in the environment, as an aggregate measure to account for the environmental impacts (caused by toxicity) of various pesticides [97]. *Pesticide pollution risk* is defined as the sum of the ratio between predicted pesticide concentrations in the environment and the predicted *no-effect concentrations*; a high pesticide pollution risk occurs when this is exceeded by three orders of magnitude (RS > 3) [97]. We define the food system boundary as the globally required reduction of pesticide application to remain within local thresholds, i.e. minimizing the exceedance of high risk and preventing the exceedance of the predicted no-effect concentrations (i.e. avoiding the introduction of additional risks to the environment). Local thresholds are based on predicted no-effect concentrations for soil, water, groundwater, and atmosphere biota [97]. Note that this estimate does not account for legacy contaminations affecting specific environmental

compartments, such as slow-recharging aquifers where contaminants turnover and biodegradation are very slow or negligible.

Global gridded pesticide application data from PEST-CHEMGRIDS [102] is used to estimate local non-cumulative pesticide environmental concentrations (PEC) using a spatial-explicit model that allows identification of locations where these concentrations transgress local thresholds [97]. The PEST-CHEMGRID covers 92 most used active ingredients from herbicides, insecticides, and fungicides at 5arcmin resolution [102]. To estimate the required reduction, we ran a global spatially explicit simulation based on [97] with various PAS application rates using application data in PEST-CHEMGRID as the baseline (i.e., a global total of about 3.6 Tg PAS yr^{-1}). With the assumption of a uniform reduction to all types of PAS, the global pesticide application reduction required to remain within local boundaries amounts to 70% (no more than about 1 Tg PAS yr^{-1}) for minimizing the exceedance of high risk and more than 90% (no more than about 0.3 Tg PAS yr^{-1}) to avoid introducing additional pollution, respectively (see **Supplementary Figure 7**).

Nonetheless, data on pesticide use is highly uncertain and likely inaccurate [103], supporting the adoption of a precautionary approach and possibly further reduction of pesticide application beyond the estimates proposed above. Furthermore, it is difficult to define a global boundary for pesticide application that allows to remain with the novel entities' boundary, while preventing risks for biosphere integrity and ecosystem functioning. Following the argumentation of [3] and [94], there is no *natural variability* of pesticide use against which a control variable can track changed on human time scales, and therefore the boundary should be set at 0% release, or limit their use to combat existing epidemics rather than use them for lowering the risk of crop yield loss. One of the major challenges for understanding Earth system impacts from pesticides, is the cumulative cocktail load and the potential interactions amongst multiple pesticides, which frustrates the setting of a boundary for the food system, despite individual safety testing, due to the large uncertainties of impact from chemical mixing in the environment.

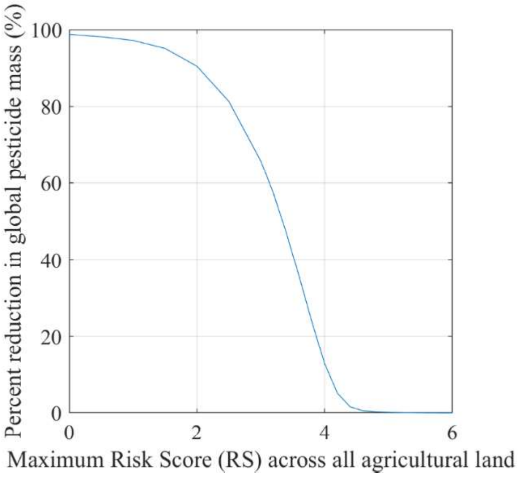

Supplementary Figure 7: Simulated response of the maximum risk score prevalence on agricultural land to the required reduction in pesticide mass (%). Limiting high-risk (RS = 3) on all agricultural land require a reduction in pesticide mass of 67%. Having no risk (RS = 0) in all agricultural lands requires a reduction of pesticide mass of close to 100%. Model approach based on [97].

Supplementary Text 9b. Antimicrobial use as an indicator for the food system contribution

Global antimicrobial use of livestock is estimated around 99-110 kilotons yr^{-1} in 2019 [104], while in aquaculture, antimicrobial use is estimated to amount 10.2 kilotons (in 2017) and expected to grow to 13.6 kilotons in 2030 [105]. In total antimicrobial use in food animals is expected to increase to around 150 kilotons yr^{-1} in 2040 (**Supplementary Table 9**). These estimates are based on the antimicrobial use intensity (mg kg^{-1}) for species (aquaculture) and livestock systems (agriculture), and country-based animal density estimates. The majority of the projected increase in antimicrobial use in the livestock sector is due to an increase in the number of animals to meet growing animal-based food demands. Shifts in production practices (towards more intensive farming operations) are responsible for a third of the projected increase. Conservative estimates of the current (2010 and 2017) antimicrobial use for food animals thus at least cover 73 kilotons yr^{-1}, while [106] suggest a total use for food animals of >130 kilotons (Table S9); and more recent estimates from [104] suggesting around 99,000-110,000 tons yr^{-1}.- The large uncertainties in the exact use rates are reflected by the uncertainty interval (95%, in between brackets) of the estimates in **Supplementary Table 10.**

Supplementary Table 10 Antimicrobial use (tonnes yr^{-1}) in agriculture, aquaculture, and all use (human + animals). Estimates between brackets are based on 95% uncertainty ranges.

	**2010 (tonnes yr^{-1})**	**2013 (tonnes yr^{-1})**	**2017 (tonnes yr^{-1})**	**2019 (tonnes yr^{-1})**	***2030 (projected)***	***2040 (projected)***	**Average intensity of use (mg kg^{-1})**

					(tonnes yr^{-1})		
**Agriculture [c] (livestock)**	63,151 (±1,560)	-	-		*105,596 (± 3,605)*		-
Chicken	-	-	-		-		2010: 148
Cattle	-	-	-		-		2010: 45
Pigs	-	-	-		-		2010: 172
**Aquaculture [b]**	-	-	10,259 (3,163 – 44,727)		*13,600 (4,193–59,295)*		2030: 164.8
**Total in food animals**	-	131,109 [a] (100,812-190,492)	-	99,414-110,777 [d]	*200,235 [a] (150,848-297,034)*	143,481 [d] (123,979–163,789)	-
**Total (human + all animals) [b]**	-	-	-		*236,757 (145,525–421,426)*		-

a) [106]; b) [105]; c) [98]; d) [104]

In response, antimicrobial resistance in the livestock sector in LMIC has rapidly increased, measured by the proportion of antimicrobial compounds with resistance higher than 50% (P50). [107] show that between 2000-2018, the P50 more than doubled in pigs (0.13 to 0.34), and almost tripled in chickens (0.15 to 0.41). For cattle, it rose from 0.12 to 0.23.

The WHO called member states to reduce antimicrobial use in the livestock sector, pointing at the risks for antimicrobial resistance transmittance to humans (and therefore do not link directly to Earth system, risks but human health risks), but did not quantify the reduction required to avoid those risks. Overall, the recommendations suggest a) an overall reduction in the use of all antimicrobial classes in food-producing animals; b) complete restriction of using antimicrobial of medical importance for growth promotion; c) complete restriction of using antimicrobials of medical importance for preventing infectious diseases that have not yet been clinically diagnosed (prophylactic use) [108]. Setting a boundary for antimicrobial use is thus challenging due to uncertainties in the environmental risks involved from increased antimicrobial resistance [109]. [106] underlines evidence of productive livestock systems that use less than half (<25 mg kg^{-1}) of the current global average antimicrobial use (50 mg kg^{-1}), which has subsequently been proposed as a target for antimicrobial use policies. Yet, it remains unclear how that target would translate to a reduction of global antimicrobial resistance; and whether this is sufficient to reduce the antimicrobial resistance levels below P50 levels (**Supplementary Figure 7**). We therefore propose a preliminary boundary for antimicrobial use in the food system, by restating the recommendation from the WHO that emphasizes a) the complete restriction of prophylactic use, while b) limiting the

average application to 25 mg kg^{-1} [106], which roughly equates halving the current use rates (average 50 mg kg^{-1}) and aggregates to a global range of 36–75 kilotons yr^{-1} (lower estimate in the range based on current animal numbers, higher end of the range based on 2040 values) In addition, a shift towards low animal-based diets can further reduce the antimicrobial use rates. However, more research is needed to quantify the required reductions in applications rates to prevent crossing the local P50 limits.

Supplementary Text 10. Methods to derive Figure2

We have integrated the present-day status of the food system and the proposed food system boundaries with the most recent PB assessment (Richardson et al., 2023) using the visual approach from Rockström et al. (2024). The PB status in our Figure 2 is based on the normalization from the PB 2.0 paper [37], where the length and colours of the wedges are normalized so that the planetary boundary and the High-risk line are uniformly projected (i.e. at equal length and colour scales). The wedges are logarithmically scaled. This deviates from the Holocene-oriented normalization approach adopted in [3]. The present-day status of food systems across the PBs (dotted overlay) is projected based on the relative contribution of food systems to the PB status (**Supplementary Table 11**), using the length of each wedge starting from the planetary boundary (so excluding the safe operating space in green) (**Supplementary Figure 8**). For the boundaries that are currently not transgressed (stratospheric ozone depletion, atmospheric aerosol loading, and ocean acidification), we use the food system boundary (red circle) as the origin to project the relative contribution of the food system. For a full account of the associated references and evidence in support of **Supplementary** Table 10, see the **Supplementary Table 1.**.

For HANPP, we have used the median of the range presented by [3,44]. For Ozone, we have used the upper end of the range of the provided agricultural contribution to N_2O emissions (55-69%), following N_2O emissions in EDGAR-FOOD (consistent with radiative forcing) [110]. For Land system change (deforestation), we used the current mean global deforestation loss (over 2001-2015) due to agriculture (permanent land use change for commodity production, and shifting cultivation) [38]. For Nitrogen, we have used to contribution of agricultural N to surface water run-off (70%). For Aerosol loading, we have used the weighted average of estimates of the contribution of food systems to anthropogenic aerosol loading (43%) (see **Supplementary Table 4**).

The food system boundary is represented in a stylized manner uniformly within the PBs, showing that these FSBs represent a share within the safe operating space.

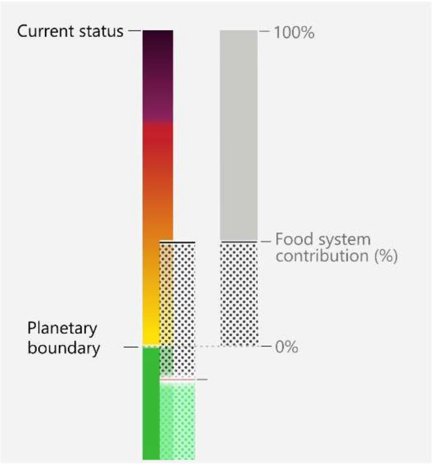

Supplementary Figure 8. Visual representation of the approach for the Food system contribution overlay in Figure 2. Note that for the three boundaries that are currently not transgressed, the origin (0%) starts at the food system boundary (pink line) rather than the planetary boundary.

Supplementary Table 11. Contribution of the food system to planetary boundary transgressions. Note that the control variable and PB status projected in [3] deviates from the control variable adopted to express the food system contribution.

Earth System Process	Control Variable in Richardson et al. (2023)	Control variable adopted in Figure 2/Table 1	Contribution of the food system (%)
**Climate change**	Atmospheric CO2 concentration (ppm CO_2)	Greenhouse gas emissions ($GtCO_2e$ yr^{-1})	±30% of total anthropogenic emissions
	Total anthropogenic radiative forcing at top-of-atmosphere	*Same as Richardson et al. (2023)*	±25 % of forcing
**Land system change**	Biome: area of forested land as the percentage of potential forest (% area remaining)	*Same as Richardson et al. (2023)*	±50% of current deforestation rate for agriculture
	Global: area of forested land as the percentage of original forest cover (%)	Area of intact land as the percentage of original cover at global level and ecoregion level (%)	±35% of total land surface used for agriculture
**Biosphere integrity**	Biosphere Functional integrity (HANPP)	*Same as Richardson et al. (2023)*	±80% of total HANPP
	Genetic diversity: E/MSY	Ecosystem Functional Integrity (intact habitat per km^2 for supporting agroecosystem functioning)	±88% on agricultural lands (by definition)
**Strato-spheric ozone depletion**	Stratospheric O_3 concentration (global average) (DU)	Same as Richardson et al. (2023), while using N_2O emissions as main ozone depleting substance (ODS)	±70% of N_2O emissions as main ODS

**Ocean acidification**	Carbonate ion concentration, average global surface ocean saturation state with respect to aragonite (Ω_{arag})	Same as Richardson et al. (2023), while using current CO_2 emissions from AFOLU as an indicator	±25% of current AFOLU CO_2 emissions
**Biogeo-chemical flows: N and P**	Phosphate:<br>Global: P flow from freshwater systems into the ocean;<br>Regional: P flow from fertilizers to erodible soils (Tg of P year−1)	Phosphorus:<br>P delivery to surface water (Tg of P yr^{-1})	±75% of total delivery
	Global: industrial and intentional fixation of N (Tg of N yr^{-1})	Nitrogen:<br>N surplus (Tg N yr^{-1})	±70% of surplus pollution
**Freshwater change**	Blue water: human induced disturbance of blue water flow	Blue water: Consumptive blue water use ($km^3 yr^{-1}$)	±70% of consumptive use
	Green water: human induced disturbance of water available to plants (% land area with deviations from preindustrial variability)	Same as Richardson et al. (2023)	±30% of transgressions [1]
**Atmospheric aerosol loading**	Interhemispheric difference in AOD	Same as Richardson et al. (2023)	±45% of anthropogenic PM2.5 concentrations
**Novel entities**	Percentage of synthetic chemical released to the environment without adequate safety testing	Proxy indicators: Pesticide Active Substances (PAS) application (Tg PAS yr^{-1}) and antimicrobial use (tons yr^{-1})	Pesticides :<br>85% of pesticides used in agriculture<br><br>Antimicrobial use:<br>73% of total antimicrobials used for food-animals

[1] It is challenging to express the contribution of food systems to global green water transgressions. Our analysis (**Supplementary Text 4**) suggests that local transgressions of green water availability in agricultural lands are similar to the global average, suggesting that agriculture does not appear a persistent, multiplying stressor on green water compared to other drivers, such as climate change, at least when globally aggregated. Preliminary experiments from the ISIMIP 3a ensemble that exclude climate change forcing using GHMs suggest that around 25-30% of the surplus transgressions rate (beyond global baseline variability) can be attributed to land use changes (dominated by agriculture) alone [111], which is what we include here as the estimate for the global contribution of food systems to the green water boundary transgression. We use the upper bound of the range to additionally account for the indirect effects of food systems' contribution to climate change, being largely responsible for the remaining freshwater boundary transgression.

Supplementary Table 12 Break-down of food system elements from Figure 1

**Agriculture**	Land use change	Drainage of organic soils
		Forest conversion
		Peat Fires
	Soils	Direct emissions
		Soil processes
	Residues	Bulk production
		Burning of residues
	Livestock	Enteric fermentation
		Grazing land area (permanent pasture and meadows)
		Manure management and application (including on pastures)
	Crop cultivation	Resource use (irrigation)
		Cropland area
	Synthetic fertilizer use	Application
**Fisheries**	Aquaculture	Excretion
		Resource use
	Wild fisheries	Vessel use
		Harvest
**Other**	Pre-production	Fertilizer manufacturing
		On-farm energy use
	Post-production	Processing
		Packaging
		Transport
		Cooking
		Retail
		Refrigeration
	Waste management	Solid waste
		Waste water

References

1. Nabuurs, G.-J. *et al.* Agriculture, Forestry and Other Land Uses (Chapter 7). in (eds Shukla, A. R. et al.) 747–860 (Cambridge University Press, Cambridge, UK and New York, NY, USA, 2022).
2. Tubiello, F. N. *et al.* Greenhouse gas emissions from food systems: building the evidence base. *Environ. Res. Lett.* **16**, 065007 (2021).
3. Richardson, K. *et al.* Earth beyond six of nine planetary boundaries. *Science Advances* **9**, eadh2458 (2023).
4. Tian, H. *et al.* Global nitrous oxide budget (1980–2020). *Earth System Science Data* **16**, 2543–2604 (2024).
5. Caleffi, S., Micha, R., Mozaffarian, D. & Springmann, M. The socio-demographic characteristics of food-related environmental impacts. Preprint at https://doi.org/10.21203/rs.3.rs-5434310/v1 (2025).
6. Rockström, J. *et al.* A roadmap for rapid decarbonization. *Science* **355**, 1269–1271 (2017).
7. Frank, S. *et al.* Agricultural non-CO2 emission reduction potential in the context of the 1.5 °C target. *Nature Clim Change* **9**, 66–72 (2019).
8. Frank, S. *et al.* Structural change as a key component for agricultural non-CO2 mitigation efforts. *Nat Commun* **9**, 1060 (2018).
9. Roe, S. *et al.* Land-based measures to mitigate climate change: Potential and feasibility by country. *Global Change Biology* **27**, 6025–6058 (2021).
10. Beillouin, D., Ben-Ari, T., Malézieux, E., Seufert, V. & Makowski, D. Positive but variable effects of crop diversification on biodiversity and ecosystem services. *Global Change Biology* **27**, 4697–4710 (2021).

11. Griscom, B. W. *et al.* Natural climate solutions. *Proceedings of the National Academy of Sciences* **114**, 11645–11650 (2017).

12. Tamburini, G. *et al.* Agricultural diversification promotes multiple ecosystem services without compromising yield. *Science Advances* **6**, (2020).

13. Lessmann, M., Ros, G. H., Young, M. D. & de Vries, W. Global variation in soil carbon sequestration potential through improved cropland management. *Global Change Biology* **28**, 1162–1177 (2022).

14. Roe, S. *et al.* Contribution of the land sector to a 1.5 °C world. *Nat. Clim. Chang.* **9**, 817–828 (2019).

15. Dietz, S., Bowen, A., Doda, B., Gambhir, A. & Warren, R. The Economics of 1.5°C Climate Change. *Annual Review of Environment and Resources* **43**, 455–480 (2018).

16. Willett, W. *et al.* Food in the Anthropocene: the EAT–Lancet Commission on healthy diets from sustainable food systems. *The Lancet* **393**, 447–492 (2019).

17. Wollenberg, E. *et al.* Reducing emissions from agriculture to meet the 2 °C target. *Glob Chang Biol* **22**, 3859–3864 (2016).

18. Smith, P. *et al.* Greenhouse gas mitigation in agriculture. *Philosophical Transactions of the Royal Society B: Biological Sciences* **363**, 789–813 (2007).

19. Forster, P. M. *et al.* Indicators of Global Climate Change 2023: annual update of key indicators of the state of the climate system and human influence. *Earth System Science Data* **16**, 2625–2658 (2024).

20. IPCC. Global Warming of 1.5°C.An IPCC Special Report on the impacts of global warming of 1.5°C above pre-industrial levels and related global greenhouse gas emission pathways, in the context of strengthening the global response to the threat

of climate change, sustainable development, and efforts to eradicate poverty. (2018).

21. Rockström, J. *et al.* Planetary Boundaries: Exploring the Safe Operating Space for Humanity. *Ecology and Society* **14**, (2009).

22. Forster, P. M. *et al.* Indicators of Global Climate Change 2022: annual update of large-scale indicators of the state of the climate system and human influence. *Earth System Science Data* **15**, 2295–2327 (2023).

23. Crippa, M., Solazzo, E., Guizzardi, D., Van Dingenen, R. & Leip, A. Air pollutant emissions from global food systems are responsible for environmental impacts, crop losses and mortality. *Nat Food* **3**, 942–956 (2022).

24. Friedlingstein, P. *et al.* Global Carbon Budget 2023. *Earth Syst. Sci. Data* **15**, 5301–5369 (2023).

25. Balasubramanian, S. *et al.* The food we eat, the air we breathe: a review of the fine particulate matter-induced air quality health impacts of the global food system. *Environ. Res. Lett.* **16**, 103004 (2021).

26. Ivanovich, C. C., Sun, T., Gordon, D. R. & Ocko, I. B. Future warming from global food consumption. *Nat. Clim. Chang.* **13**, 297–302 (2023).

27. Springmann, M. *et al.* The global and regional air quality impacts of dietary change. *Nat Commun* **14**, 6227 (2023).

28. Crippa, M. *et al.* GHG emissions of all world countries. *JRC Publications Repository* https://publications.jrc.ec.europa.eu/repository/handle/JRC138862 (2024) doi:10.2760/4002897.

29. Klein Goldewijk, K., Beusen, A., Doelman, J. & Stehfest, E. Anthropogenic land use estimates for the Holocene – HYDE 3.2. *Earth System Science Data* **9**, 927–953 (2017).

30. FAO. Land statistics 2001–2022. (2024).

31. Potapov, P. *et al.* Global maps of cropland extent and change show accelerated cropland expansion in the twenty-first century. *Nat Food* **3**, 19–28 (2022).

32. Tubiello, F. N. *et al.* Measuring the world's cropland area. *Nat Food* **4**, 30–32 (2023).

33. Rockström, J. *et al.* Safe and just Earth system boundaries. *Nature* **619**, 102–111 (2023).

34. DeClerck, F. A. J. *et al.* A Whole Earth Approach to Nature-Positive Food: Biodiversity and Agriculture. in *Science and Innovations for Food Systems Transformation* (eds von Braun, J., Afsana, K., Fresco, L. O. & Hassan, M. H. A.) 469–496 (Springer International Publishing, Cham, 2023). doi:10.1007/978-3-031-15703-5_25.

35. Gerten, D. *et al.* Feeding ten billion people is possible within four terrestrial planetary boundaries. *Nat Sustain* **3**, 200–208 (2020).

36. Springmann, M. *et al.* Options for keeping the food system within environmental limits. *Nature* **562**, 519–525 (2018).

37. Steffen, W. *et al.* Planetary boundaries: Guiding human development on a changing planet. *Science* **347**, 1259855 (2015).

38. Curtis, P. G., Slay, C. M., Harris, N. L., Tyukavina, A. & Hansen, M. C. Classifying drivers of global forest loss. *Science* **361**, 1108–1111 (2018).

39. Pendrill, F. *et al.* Disentangling the numbers behind agriculture-driven tropical deforestation. *Science* **377**, eabm9267 (2022).

40. Olson, D. M. *et al.* Terrestrial Ecoregions of the World: A New Map of Life on Earth: A new global map of terrestrial ecoregions provides an innovative tool for conserving biodiversity. *BioScience* **51**, 933–938 (2001).

41. DeClerck, F., Jones, S., Estrada-Carmona, N. & Fremier, A. *Spare Half, Share the Rest: A Revised Planetary Boundary for Biodiversity Intactness and Integrity*. (2021). doi:10.21203/rs.3.rs-355772/v1.

42. Dinerstein, E. *et al.* An Ecoregion-Based Approach to Protecting Half the Terrestrial Realm. *BioScience* **67**, 534–545 (2017).

43. Haberl, H. *et al.* Quantifying and mapping the human appropriation of net primary production in earth's terrestrial ecosystems. *Proceedings of the National Academy of Sciences* **104**, 12942–12947 (2007).

44. Stenzel, F. *et al.* biospheremetrics v1.0.2: an R package to calculate two complementary terrestrial biosphere integrity indicators – human colonization of the biosphere (BioCol) and risk of ecosystem destabilization (EcoRisk). *Geoscientific Model Development* **17**, 3235–3258 (2024).

45. Krausmann, F. *et al.* Global human appropriation of net primary production doubled in the 20th century. *Proceedings of the National Academy of Sciences* **110**, 10324–10329 (2013).

46. Heinke, J. *et al.* Water Use in Global Livestock Production—Opportunities and Constraints for Increasing Water Productivity. *Water Resources Research* **56**, e2019WR026995 (2020).

47. Mohamed, A. *et al.* Securing Nature's Contributions to People requires at least 20%–25% (semi-)natural habitat in human-modified landscapes. *One Earth* **7**, 59–71 (2024).

48. Mohamed, A. *et al.* Securing Nature's Contributions to People requires at least 20%–25% (semi-)natural habitat in human-modified landscapes. *One Earth* **7**, 59–71 (2024).

49. Chaplin-Kramer, R. *et al.* Global modeling of nature's contributions to people. *Science* **366**, 255–258 (2019).

50. Gerten, D. *et al.* Towards a revised planetary boundary for consumptive freshwater use: role of environmental flow requirements. *Current Opinion in Environmental Sustainability* **5**, 551–558 (2013).

51. Jägermeyr, J., Pastor, A., Biemans, H. & Gerten, D. Reconciling irrigated food production with environmental flows for Sustainable Development Goals implementation. *Nat Commun* **8**, 15900 (2017).

52. Porkka, M. *et al.* Notable shifts beyond pre-industrial streamflow and soil moisture conditions transgress the planetary boundary for freshwater change. *Nat Water* **2**, 262–273 (2024).

53. Pastor, A. V. *et al.* Understanding the transgression of global and regional freshwater planetary boundaries. *Philosophical Transactions of the Royal Society A: Mathematical, Physical and Engineering Sciences* **380**, 20210294 (2022).

54. McDermid, S. *et al.* Irrigation in the Earth system. *Nat Rev Earth Environ* 1–19 (2023) doi:10.1038/s43017-023-00438-5.

55. Campbell, B. M. *et al.* Agriculture production as a major driver of the Earth system exceeding planetary boundaries. *Ecology and Society* **22**, (2017).

56. Shiklomanov, I. & Rodda, J. World Water Resources at the Beginning of the Twenty-First Century. **13**, (2004).

57. de Graaf, I. E. M., Gleeson, T., (Rens) van Beek, L. P. H., Sutanudjaja, E. H. & Bierkens, M. F. P. Environmental flow limits to global groundwater pumping. *Nature* **574**, 90–94 (2019).

58. Stewart-Koster, B. *et al.* Living within the safe and just Earth system boundaries for blue water. *Nat Sustain* 1–11 (2023) doi:10.1038/s41893-023-01247-w.

59. Schyns, J. F., Hoekstra, A. Y., Booij, M. J., Hogeboom, R. J. & Mekonnen, M. M. Limits to the world's green water resources for food, feed, fiber, timber, and bioenergy. *Proceedings of the National Academy of Sciences* **116**, 4893–4898 (2019).

60. Hoff, H. *et al.* Greening the global water system. *J. Hydrol.* **384**, 177–186 (2010).

61. Rost, S. *et al.* Agricultural green and blue water consumption and its influence on the global water system. *Water Resources Research* **44**, (2008).

62. Schaphoff, S. *et al.* LPJmL4 – a dynamic global vegetation model with managed land – Part 1: Model description. *Geoscientific Model Development* **11**, 1343–1375 (2018).

63. Gerten, D. *et al.* A software package for assessing terrestrial planetary boundaries. *One Earth* 101341 (2025) doi:10.1016/j.oneear.2025.101341.

64. Gerten, D. *et al.* A Software Package for Assessing Terrestrial Planetary Boundaries. SSRN Scholarly Paper at https://doi.org/10.2139/ssrn.4890102 (2024).

65. Lai, E. N., Wang-Erlandsson, L., Virkki, V., Porkka, M. & van der Ent, R. J. Root zone soil moisture in over 25 % of global land permanently beyond pre-industrial variability as early as 2050 without climate policy. *Hydrology and Earth System Sciences* **27**, 3999–4018 (2023).

66. Mekonnen, M. M. & Hoekstra, A. Y. The green, blue and grey water footprint of crops and derived crop products. *Hydrology and Earth System Sciences* **15**, 1577–1600 (2011).

67. Siebert, S. & Döll, P. Quantifying blue and green virtual water contents in global crop production as well as potential production losses without irrigation. *Journal of Hydrology* **384**, 198–217 (2010).

68. Hanasaki, N., Inuzuka, T., Kanae, S. & Oki, T. An estimation of global virtual water flow and sources of water withdrawal for major crops and livestock products using a global hydrological model. *J. Hydrol.* **384**, 232–244 (2010).

69. Oki, T. & Kanae, S. Global Hydrological Cycles and World Water Resources. *Science* **313**, 1068–1072 (2006).

70. Wada, Y., van Beek, L. P. H. & Bierkens, M. F. P. Modelling global water stress of the recent past: on the relative importance of trends in water demand and climate variability. *Hydrology and Earth System Sciences* **15**, 3785–3808 (2011).

71. Qin, Y. *et al.* Flexibility and intensity of global water use. *Nat Sustain* **2**, 515–523 (2019).

72. de Vries, W., Kros, J., Kroeze, C. & Seitzinger, S. P. Assessing planetary and regional nitrogen boundaries related to food security and adverse environmental impacts. *Current Opinion in Environmental Sustainability* **5**, 392–402 (2013).

73. Schulte-Uebbing, L. F., Beusen, A. H. W., Bouwman, A. F. & de Vries, W. From planetary to regional boundaries for agricultural nitrogen pollution. *Nature* **610**, 507–512 (2022).

74. de Vries, W., Schulte-Uebbing, L. F., Beusen, A. H. W. & Te Wierik, S. A. Revisiting planetary boundaries for nitrogen and phosphorus. *Manuscript submitted for publication* (2024).

75. Schulte-Uebbing, L. F., Beusen, A. H. W., Bouwman, A. F. & de Vries, W. From planetary to regional boundaries for agricultural nitrogen pollution. *Nature* **610**, 507–512 (2022).

76. Yuan, Z. *et al.* Human Perturbation of the Global Phosphorus Cycle: Changes and Consequences. *Environ. Sci. Technol.* **52**, 2438–2450 (2018).

77. Poikane, S. *et al.* Nutrient criteria for surface waters under the European Water Framework Directive: Current state-of-the-art, challenges and future outlook. *Science of The Total Environment* **695**, 133888 (2019).

78. Poikane, S. *et al.* Deriving nutrient criteria to support 'good' ecological status in European lakes: An empirically based approach to linking ecology and management. *Science of The Total Environment* **650**, 2074–2084 (2019).

79. Beusen, A. H. W. *et al.* Exploring river nitrogen and phosphorus loading and export to global coastal waters in the Shared Socio-economic pathways. *Global Environmental Change* **72**, 102426 (2022).

80. Schwede, D. B. *et al.* Spatial variation of modelled total, dry and wet nitrogen deposition to forests at global scale. *Environmental Pollution* **243**, 1287–1301 (2018).

81. Bouwman, A. F., Beusen, A. H. W., Doelman, J. C., Stehfest, E. & Westhoek, H. Impact of lifestyle, human diet and nutrient use efficiency in food production on eutrophication of global aquifers and surface waters. *Global Environmental Change* **87**, 102874 (2024).

82. Ravishankara, A. R., Daniel, J. S. & Portmann, R. W. Nitrous Oxide (N2O): The Dominant Ozone-Depleting Substance Emitted in the 21st Century. *Science* **326**, 123–125 (2009).

83. Gruber, N. *et al.* Trends and variability in the ocean carbon sink. *Nat Rev Earth Environ* **4**, 119–134 (2023).

84. Dhakal, S. *et al. Chapter 2: Emissions Trends and Drivers*. doi: 10.1017/9781009157926.004 (2022).

85. Gruber, N. Warming up, turning sour, losing breath: ocean biogeochemistry under global change. *Philosophical Transactions of the Royal Society A: Mathematical, Physical and Engineering Sciences* **369**, 1980–1996 (2011).

86. Kessouri, F. *et al.* Coastal eutrophication drives acidification, oxygen loss, and ecosystem change in a major oceanic upwelling system. *Proceedings of the National Academy of Sciences* **118**, e2018856118 (2021).

87. Vogel, A. *et al.* Uncertainty in Aerosol Optical Depth From Modern Aerosol-Climate Models, Reanalyses, and Satellite Products. *Journal of Geophysical Research: Atmospheres* **127**, e2021JD035483 (2022).

88. Lambert, A. *et al.* Dust Impacts of Rapid Agricultural Expansion on the Great Plains. *Geophysical Research Letters* **47**, e2020GL090347 (2020).

89. Bauer, S. E., Tsigaridis, K. & Miller, R. Significant atmospheric aerosol pollution caused by world food cultivation. *Geophysical Research Letters* **43**, 5394–5400 (2016).

90. Erisman, J. W. & Schaap, M. The need for ammonia abatement with respect to secondary PM reductions in Europe. *Environmental Pollution* **129**, 159–163 (2004).

91. Lelieveld, J., Evans, J. S., Fnais, M., Giannadaki, D. & Pozzer, A. The contribution of outdoor air pollution sources to premature mortality on a global scale. *Nature* **525**, 367–371 (2015).

92. Gatto, A. & Chepeliev, M. Reducing global food loss and waste could improve air quality and lower the risk of premature mortality. *Environ. Res. Lett.* **19**, 014080 (2024).

93. Burton, C. *et al.* Global burned area increasingly explained by climate change. *Nat. Clim. Chang.* 1–7 (2024) doi:10.1038/s41558-024-02140-w.

94. Persson, L. *et al.* Outside the Safe Operating Space of the Planetary Boundary for Novel Entities. *Environ. Sci. Technol.* **56**, 1510–1521 (2022).

95. Betarbet, R. *et al.* Chronic systemic pesticide exposure reproduces features of Parkinson's disease. *Nat Neurosci* **3**, 1301–1306 (2000).

96. Okeke, I. N. *et al.* The scope of the antimicrobial resistance challenge. *The Lancet* **403**, 2426–2438 (2024).

97. Tang, F. H. M., Lenzen, M., McBratney, A. & Maggi, F. Risk of pesticide pollution at the global scale. *Nat. Geosci.* **14**, 206–210 (2021).

98. Van Boeckel, T. P. *et al.* Global trends in antimicrobial use in food animals. *Proceedings of the National Academy of Sciences* **112**, 5649–5654 (2015).

99. Cassou, E. Pesticides. *World Bank* https://documents.worldbank.org/en/publication/documents-reports/documentdetail/689281521218090562/Pesticides (2018).

100. Schulz, R., Bub, S., Petschick, L. L., Stehle, S. & Wolfram, J. Applied pesticide toxicity shifts toward plants and invertebrates, even in GM crops. *Science* **372**, 81–84 (2021).

101. Bhandari, G., Atreya, K., Scheepers, P. T. J. & Geissen, V. Concentration and distribution of pesticide residues in soil: Non-dietary human health risk assessment. *Chemosphere* **253**, 126594 (2020).

102. Maggi, F., Tang, F. H. M., la Cecilia, D. & McBratney, A. PEST-CHEMGRIDS, global gridded maps of the top 20 crop-specific pesticide application rates from 2015 to 2025. *Sci Data* **6**, 170 (2019).

103. Shattuck, A., Werner, M., Mempel, F., Dunivin, Z. & Galt, R. Global pesticide use and trade database (GloPUT): New estimates show pesticide use trends in low-income countries substantially underestimated. *Global Environmental Change* **81**, 102693 (2023).

104. Acosta, A. *et al.* The future of antibiotic use in livestock. *Nat Commun* **16**, 2469 (2025).

105. Schar, D., Klein, E. Y., Laxminarayan, R., Gilbert, M. & Van Boeckel, T. P. Global trends in antimicrobial use in aquaculture. *Sci Rep* **10**, 21878 (2020).

106. Van Boeckel, T. P. *et al.* Reducing antimicrobial use in food animals. *Science* **357**, 1350–1352 (2017).

107. Van Boeckel, T. P. *et al.* Global trends in antimicrobial resistance in animals in low- and middle-income countries. *Science* **365**, eaaw1944 (2019).

108. World Health Organization. *WHO Guidelines on Use of Medically Important Antimicrobials in Food-Producing Animals*. (World Health Organization, Geneva, 2017).

109. Larsson, D. G. J., Gaze, W. H., Laxminarayan, R. & Topp, E. AMR, One Health and the environment. *Nat Microbiol* **8**, 754–755 (2023).

110. Crippa, M. *et al.* Food systems are responsible for a third of global anthropogenic GHG emissions. *Nat Food* **2**, 198–209 (2021).

111. Virkki, V., Andersen, L. S., Wierik, S. te, Gerten, D. & Porkka, M. Regionally divergent drivers behind transgressions of the freshwater change planetary boundary. (2025).
